# CRISPR/Cas9 mediated genetic resource for unknown kinase and phosphatase genes in *Drosophila*

**DOI:** 10.1038/s41598-020-64253-4

**Published:** 2020-04-30

**Authors:** Menghua Wu, Xuedi Zhang, Wei Wei, Li Long, Sainan An, Guanjun Gao

**Affiliations:** 1 School of Life Sciences, Tsinghua University, Beijing, 100084 P.R. China; 2 School of Life Science and Technology, ShanghaiTech University, Shanghai, 201210 P.R. China; 3 National Institute of Biological Sciences, Beijing, 102206 P.R. China

**Keywords:** Developmental biology, Genetics

## Abstract

Kinases and phosphatases are crucial for cellular processes and animal development. Various sets of resources in *Drosophila* have contributed significantly to the identification of kinases, phosphatases and their regulators. However, there are still many kinases, phosphatases and associate genes with unknown functions in the *Drosophila* genome. In this study, we utilized a CRISPR/Cas9 strategy to generate stable mutants for these unknown kinases, phosphatases and associate factors in *Drosophila*. For all the 156 unknown gene loci, we totally obtained 385 mutant alleles of 105 candidates, with 18 failure due to low efficiency of selected gRNAs and other 33 failure due to few recovered F0, which indicated high probability of lethal genes. From all the 105 mutated genes, we observed 9 whose mutants were lethal and another 4 sterile, most of which with human orthologs referred in OMIM, representing their huge value for human disease research. Here, we deliver these mutants as an open resource for more interesting studies.

## Introduction

Phosphorylation, the most common post-translational modification (PTMs) of proteins, is involved in multiple biological processes in eukaryotic organisms. Kinases and phosphatases collaborate to regulate the levels of this modification^1,2^, and mutations of these always act as causal factors in human diseases^3,4^. Thus, a deep exploration of kinase and phosphatase genes function will aid in the study of human diseases in the clinical^5^. *Drosophila melanogaster* is an ideal system for the dissection of kinase and phosphatase gene function because of its high gene conservation with the human genome and low gene redundancy in its own genome^6^. P-element-mediated insertion and RNAi both are well-established methods for gene function analysis in *Drosophila*, and there are abundant genetic resources for both of these systems^7,8^. In fact, *Drosophila* genetic screening using these resources has contributed significantly to the identification of kinases, phosphatases, and their regulators^9–11^. However, there are still many unknown functional genes with predicted kinase and phosphatase domains in the *Drosophila* genome, and we sought to develop a high-throughput gene-targeting strategy to aid with characterizing them.

The emergence of CRISPR/Cas9 made it possible for us to manipulate high-throughput mutagenesis in *Drosophila*^12–15^. In our previous work, the CRISPR/Cas9 mediated mutagenesis frequency even could reach 100% in some cases^15^. Besides, we also applied this CRISPR/Cas9 system to a *Drosophila* testis specific lncRNA knockout project and demonstrated its high-throughput application^16^. In this study, we carried out another large-scale mutagenesis for 156 unknown kinase and phosphatase genes and finally obtained 385 mutant alleles for 105 individual genes with 33 high potential lethal genes. Using these mutants, we uncovered several functional kinases and phosphatases, providing valuable genetic resource for phosphorylation clinical research.

## Results

### Unknown kinase, phosphatase and associate factors in *Drosophila* genome

**Figure 1:**
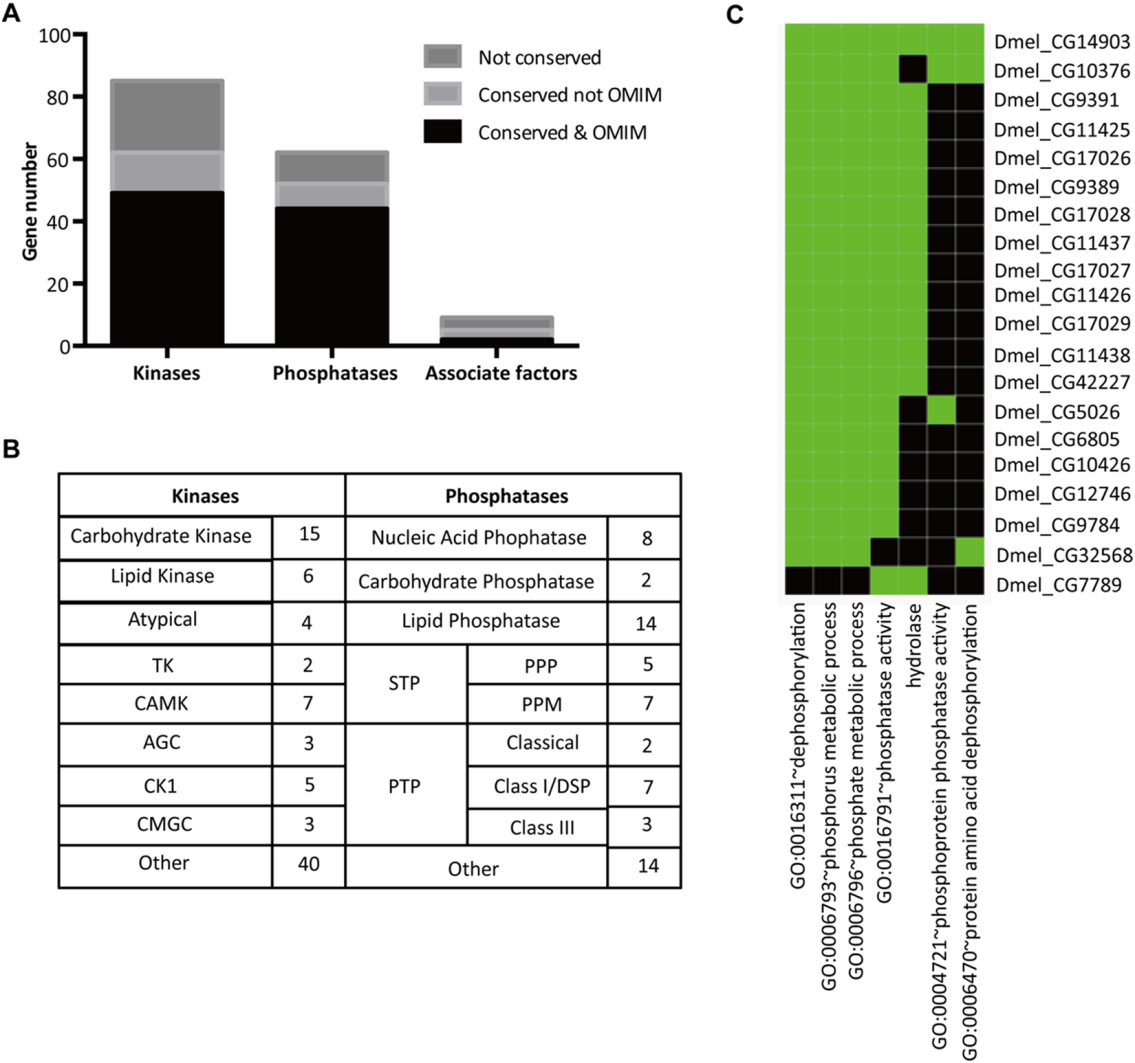
Unknown kinases, phosphatases and associate factors in *Drosophila* genome. (**A**) Graphical demonstration of the conservation with human of the candidates. Conservation was considered when a high confidence human ortholog was predicted via DIOPT (score > 3)^26^. (**B**) Gene group category of the candidates. (**C**) Gene ontology analysis of the candidates. Gene-term enrichment was identified using the DAVID functional annotation tool^27^. Green box, corresponding gene-term positively reported (mainly by prediction). Black box, corresponding gene-term not reported yet.

*Drosophila* genome encodes 376 kinases, 159 phosphatases and 27 associate factors that associated with these enzymes, such as cyclins and regulatory subunits^2,9,17^. After thoroughly searching in FlyBase, we compiled a list of 85 kinases, 63 phosphatases and 9 associate factors with ambiguous functional annotations (Supplementary Table 1). This accounts for 23%, 40% and 33% of all kinases, phosphatases and associate factors encoded in the *Drosophila* genome, respectively. Among all these 156 unknown kinase, phosphatase and associate genes, we found 70% (119/156) were conserved with human genome, 90% (95/119) of which human orthologs were linked to human disease in Online Mendelian Inheritance in Man (OMIM). Gene group category divided these candidates into several families. Gene ontology analysis showed they might be involved in multiple developmental processes (Fig. 1). These all indicated their important functions, while perhaps due to current limited resources, their functions have not been defined yet. Thus, we planned to generate stable mutants to define the function of all these 156 unknown kinases and phosphatases.

### CRISPR/Cas9 mediated mutagenesis of the unknown candidates

**Figure 2:**
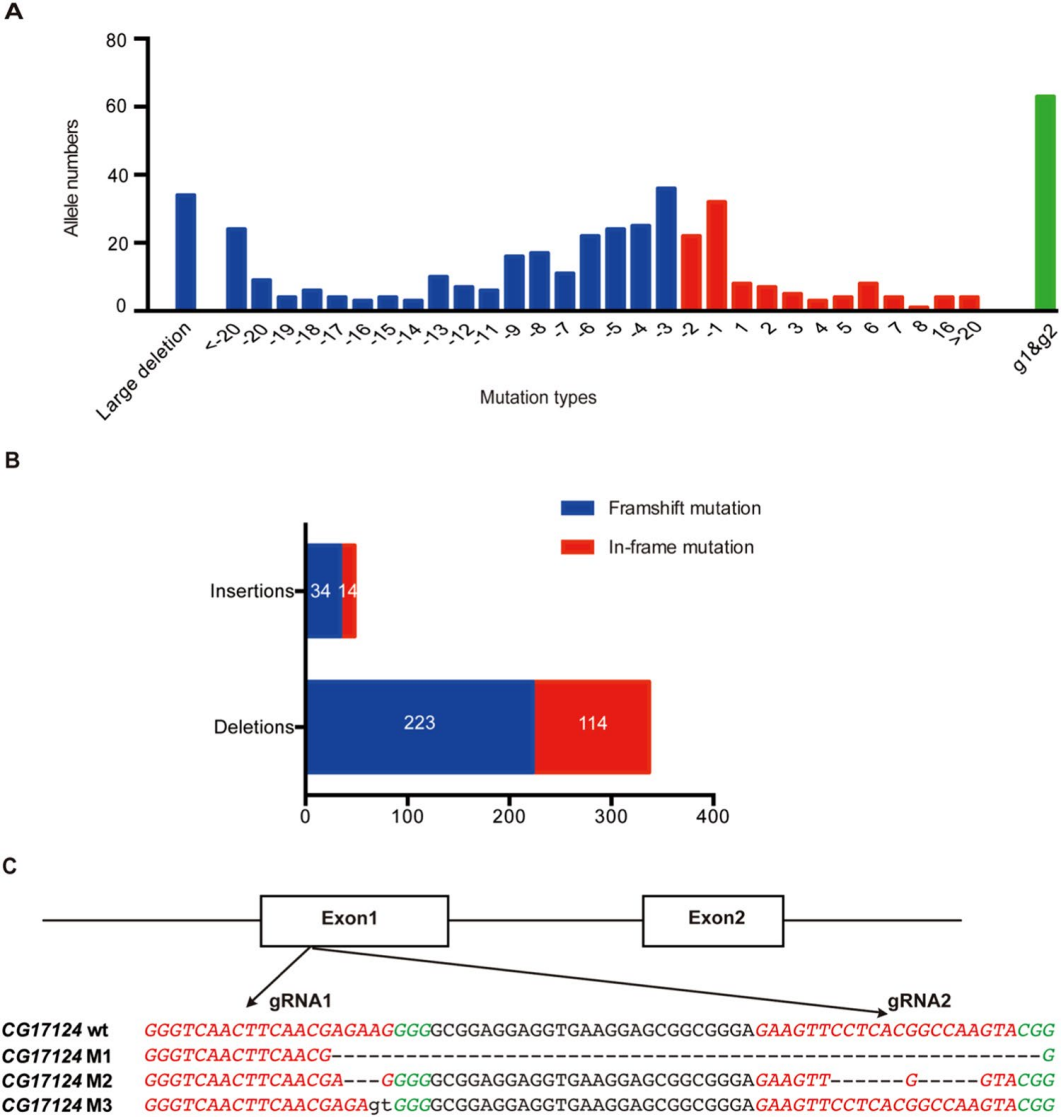
CRISPR/Cas9 mediated mutagenesis of the unknown candidates. (**A**) Mutations of all the 385 mutant alleles classified by deletions and insertions. Blue bars, deletions. Red bars, insertions. Green bars, two gRNAs working simultaneously, including both deletions and insertions. (**B**) Mutations of all the 385 mutant alleles classified by in-frame or frameshift of the coding sequence. (**C**) Exampled mutation types in one single gene. The DNA sequence was obtained from the flybase website (flybase.org) and the alignment was performed by CLUSTALW. Red area, predicted gRNA recognition site. Green area, PAM sequence. M1, g1 to g2 large deletion. M2, g1 and g2 disruption. M3, g1 disruption.

To ensure the mutagenesis efficiency and completely disrupt the gene function, we designed two gRNAs per gene around the translational start codon or at least before 1/3 of the coding sequence when carrying out mutagenesis according to the standard CRISPR/Cas9 strategy. In case that these unknown genes could be lethal when mutated, we used different FRT flies for microinjection according to their genomic distribution in order to perform tissue-specific mosaic analysis. Through separate operation on the five individual FRT groups, we finally obtained 385 mutant alleles for 105 genes from all the 156 candidates and another 33 s failed due to few F0, indicating high probability of lethal genes, which we called potential lethal genes. While for the remnant 18 failure, we found they were nothing with the gRNA working efficiency, length, sequence specificity or PAM sequence specificity (Supplementary Figure 1), which perhaps mainly caused by limited detected F0/F1. Consistent with results from other organisms^18^, we also detected insertions, deletions including frameshift and in-frame mutations among the 385 alleles (Fig. 2, Supplementary Note 1), in which case there were 11 genes of the 105 with only in-frame mutants in the resource. Of the 385 mutant alleles, we randomly chose 6 mutants to detect the mRNAs of the corresponding mutant genes using qRT-PCR. As expected, we observed reduced mRNAs to different degrees of detected genes (Supplementary Figure 2), demonstrating our resource with corresponding gene deficiency.

### Obvious phenotypes in the CRISPR/Cas9 resource

Considering complicated functions of the kinases and phosphatases, we performed simple lethality and sterility screening for the resource only in this study. Taken together, we observed 9 genes with lethal mutants, 1 gene with male sterile mutants and 3 genes with female sterile mutants, these genes were predicted involved in various developmental processes (Table 1). To be recommended, of the 3 female sterile events, mutations of *CG3608* were all in-frame styles, implicating the importance of the missed amino acids in the mutants, meanwhile, we could not exclude the possibility that there were off-targeting effects in the mutants. The same situation occurred in the rescue experiments for the phenotypic mutants. Of the 13 phenotypic mutants, we totally generate 4 transgenic lines including *CG33671*, *CG17028*, *CG14305* and *CG15743* to rescue the corresponding mutants, but we found the *CG17028* transgenic lines could not rescue the lethality of *CG17028* mutants, which might be caused by ectopic transgenes or off-targeting effects. Besides these, we also observed other abnormal lethal events in the resource, such as *CG12229* and *CG17027*, with different lethality between similar or same genotypic mutants, even one line of *CG17028*, without any mutation detected near the gRNA recognition sites (Supplementary Figure 3, Supplementary Note 2). Anyway, despite uncertainty of the total resource, we could define more certain phenotypes of unknown candidates such as lethality and sterility. We look forward to more interesting phenotypes in our resource.

**Table 1:** Obvious phenotypes in the CRISPR/Cas9 resource.

Gene symbol	Human ortholog(*OMIM)	Phenotype	Predicted function
*CG34380*	DDR1*,2*	Lethal	Peptidyl-tyrosine phosphorylation
*CG9222*	TSSK1B*,2*,4*	Lethal	Protein serine/threonine kinase
*CG7236*	CDKL1*,2*,3*,5*	Lethal	Mitotic cytokinesis
*CG33671*	MVK*	Lethal^a^	Isoprenoid biosynthetic process
*CG10738*	NPR1*,2*	Lethal	Positive regulation of cell proliferation
*CG6767*	PRPS1*, S1L1*	Lethal	Imaginal-disc drived wing morphogenesis
*CG17028*	IMPA1*,2*	Lethal ^b^	Insitol phosphate dephosphorylation
*CG9784*	*——*	Lethal	Phosphatidylinositol dephosphorylation
*CG34455*	*——*	Lethal	Cellular response to monosaccharide
*CG14305*	TSSK1B*,2*,3*,6*	Male sterile^a^	Protein serine/threonine kinase
*CG3608*	ADCK1*	Female sterile^#^	Positive regulation of multicellular organism growth
*CG4041*	*TBCK*	Female sterile	Golgi organization
*CG15743*	IMPAD1*, UBE2W*	Female sterile^a^	Phosphatidylinositol biosynthetic process

^a^The lethality phenotype of *CG33671* and male sterility phenotype of *CG14305* both can be rescued by their own original genomic transcripts. ^b^The lethality phenotype of *CG17028* cannot be rescued by its original genomic transcript. ^#^The female sterility phenotype of *CG3608* was observed from the in-frame mutant alleles, because we did not obtain frameshift mutant alleles for *CG3608* in our resource.

## Discussion

Our work in this study delivered a valuable genetic resource for unknown kinase, phosphatase and associate genes in *Drosophlia*. Using this resource, we can carry out lots of meaningful and interesting screenings besides of lethality and fertility. Of the lethal and sterile genes identified in this study (Table 1), we re-searched for gene function and found some of them had been experimentally investigated, such as CG34380, proved to be required for normal thermal nociception in a RNAi-based screening study^19^; CG6767, proved to affect olfactory behavior using P-element insertional mutagenesis together with targeted RNAi^20^; and CG34455, prove to encode a pyridoxal kinase and contribute to chromosome integrity and glucose homeostasis through mutation isolated from EMS-induced late lethals^21^, our identification of which providing direct evidence for their important roles in multiple developmental processes and efficient genetic tools to address associated issues.

During the generation of this resource, we observed three insights into large-scale CRISPR/Cas9 application. First, the regular CRISPR/Cas9 system is not as well suited to screening lethal genes by high-throughput mutagenesis as it is to viable genes *in vivo*, and it was completely unable to outperform genetic screens as observed in cell lines^22^. For the 33 s failure due to few F0s in the resource, more efforts were needed to get stable mutant lines through regular CRISPR/Cas9.

Second, based on roughly calculation between the mutation yield and gRNA working efficiency, length, sequence, PAM sequence, we found these factors seem do nothing with the mutation yield, except for a 19-nt GGG gRNA pattern, which perhaps caused by the single 17-G (Supplementary Figure 1C-D), consistent with results previously described^23^.

Finally, we observed some potential off-targeting effects during the generation of our resource. In addition to the confirmed abnormality, the abnormal lethality that could not be rescued for *CG17028* hinted at the increasing off-targeting possibility of the remaining viable alleles. Regretfully, we sequenced only about 200 bp near the gRNA recognition sites of these abnormal lethal lines, thus we cannot exclude the possibility that there were different mutations between the lethal lines and viable lines for the same gene in the target gene region far away from the gRNA recognition sites^24^, another side-effects caused by CRISPR/Cas9 treatment. Regardless, our results present there did exist off-targeting or side-effects during the CRISPR/Cas9 mediated mutagenesis, suggesting the need for more specific CRISPR/Cas9^25^ or more efficient and precise genome editing methods for gene therapy of diseases.

## Methods

### Fly strains

w^1118^, FM7a, Sco/Cyo, Sb/TM6B, Sco/Cyo; Sb/TM6B were stocks from our own lab (ShanghaiTech University, China); FRT19A was from Jose Carlos Pastor-Pareja’s lab (Tsinghua University, China); FRT40A, FRT42D, FRT79D were from Ting Xie’s lab (Tsinghua University, China); FRT82B was from Renjie Jiao’s lab (Institute of Biophysics, CAS, China); attP40, attP2 were from Xiaolin Bi’s lab (Dalian Medical University, China). All flies were cultured at 25 °C.

### gRNA *in vitro* transcription and microinjection

gRNAs were transcribed as previously described^15^, then mixed with Cas9 mRNA at a final concentration of 500 ng/ul and injected into different FRT flies.

### T7EI assay and mutation identification

Dead larvae or single flies were squashed in 30 uL of squashing buffer containing 10 mM Tris-Hcl (PH 8.0), 1 mM EDTA, 25 mM NaCl, 1 mg/mL proteinase K (Takara, Beijing, China), and incubated at 37 °C for 1 h, followed by heating to 95 °C for 2 min and used as PCR templates. PCR was performed in a 2 x Taq MasterMix (Aidlab Biotech, Beijing, China) under standard PCR procedure, with primers 100–200 bp away from the gRNA targeting site. The PCR products were then digested with T7 Endonuclease I (Viewsolid Biotech, Beijing, China), and T7EI positive F1 PCR products were sequenced for mutants.

### Fertility screening

Every three males or virgin-females of our resource with viable homozygous were crossed with w^1118^ virgin-females or males. Parents were kept for 7 days and discarded. Then adult flies of each cross were counted for fertility of the viable resource. The ones with no offspring were defined as sterile.

### Plasmid constructions and generation of transgenic flies

Full length genomic fragments were amplified using the following primers: *CG33671*-KpnIF, 5′ GATATCTAggtaccCCTTAGGCTTCGTGGGACTCTTA 3′, *CG33671*-NotIR, 5′ GTACATgcggccgcTCTGTCAGTGTCGTGGCTTGGTT 3′; *CG17028*-KpnIF,5′TACATGTAggtaccAGGAGGGCTATCAGAAGGCAAAG 3′, *CG17028*-NotIR, 5′ GTACATgcggccgcGCCCAGTTTCGTTGTGAAGAGC 3′; *CG14305*-NotIF, 5′ TGACATgcggccgcTTGTGCTGCGTGCGTGTAAAT 3′, 1 *CG14305*-KpnIR, 5′ GATCTAggtaccACTTATTGCGAGGAGACTGCC 3′; *CG15743*-KpnIF, 5′ GATATCTAggtaccGAGTGAGGAGCAAGACAGACGAAA 3′, and *CG15743*-NotIR, 5′ TAGTACTTgcggccgcCAGTTGGTTATCTGCTGCTCATCG 3′. PCR products were then cut with KpnI and NotI, and then cloned into the pUAST-attB plasmid^16^. pUAST-attB-*CG33671* and pUAST-attB-*CG15743* were introduced into the attP2 site by the phiC31 integration method, and the pUAST-attB-*CG17028* and pUAST-attB-*CG14305* were introduced into the attP40 site.

## Data availability

The flies will be available by mail by contacting the corresponding author, Dr. Gao, who will maintain the stocks in the laboratory.

## Supplementary information

Supplementary Information.

Supplementary Table 1
