# Supplementary material for: CRISPR/Cas9 mediated genetic resource for unknown kinase and phosphatase genes in Drosophila
Source: Sci Rep. 2020 Apr 30;10:7383. doi: 10.1038/s41598-020-64253-4 (PMC7193564; doi:10.1038/s41598-020-64253-4)
Supplement: Supplementary file 1 — Supplementary Information. [file 41598_2020_64253_MOESM1_ESM.pdf]

CRISPR/Cas9-mediated genetic resource for unknown kinase and phosphatase genes in *Drosophila*

Menghua Wu[1,2], Xuedi Zhang[2], Wei Wei[1], Li Long[1], Sainan An[3] and Guanjun Gao[2,1,*]

[1] School of Life Sciences, Tsinghua University, Beijing, 100084, PR China
[2] School of Life Science and Technology, ShanghaiTech University, Shanghai, 201210, PR China
[3] National Institute of Biological Sciences, Beijing, 102206, PR China
* Corresponding：gaogj@shanghaitech.edu.cn

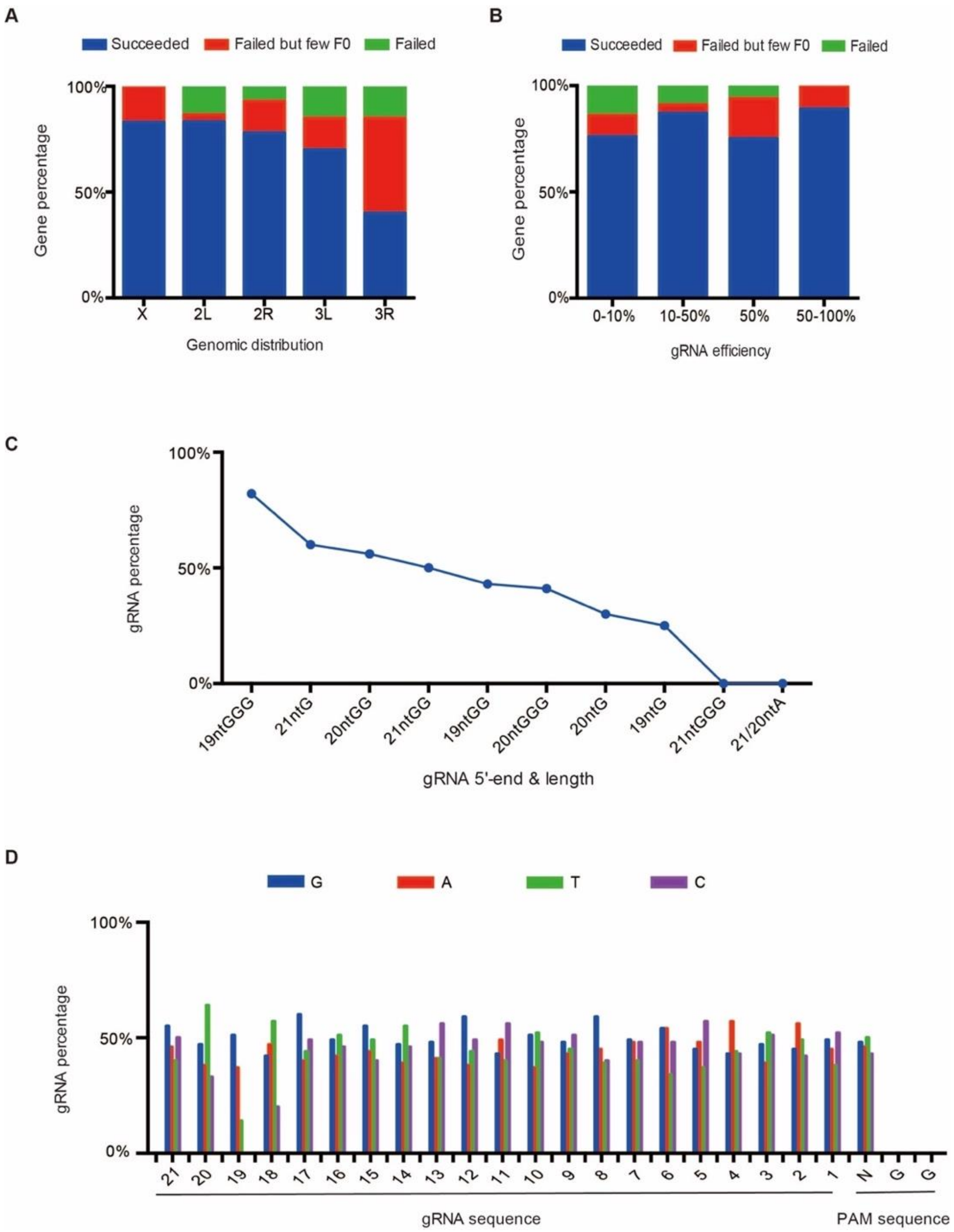

Supplementary Figure 1. CRISPR/Cas9-mediated mutagenesis efficiency. (A) Mutant yield of different genomic subgroups classified by whether stable mutants were successfully generated. The high percentage of too few F0s in group 3R might be due to the injection of FRT82B flies. (B) The gene percentage of different levels of gRNA efficiency by whether stable mutants were generated. The gRNA efficiency was defined based on a T7EI assay activity with dead injected F0 larvae and classified by different levels of activity (Supplementary Figure 1C). (C) The gRNA percentage of different 5'-end "G"s by whether mutations at the gRNA recognition site were detected. (D) The gRNA percentage at different positions in the gRNA and PAM sequence by whether mutations at the gRNA recognition site were detected. Twenty-one nt of the gRNA sequence was calculated, and the original genomic sequence was added in the case of 19-20 nt gRNA, with the data at positions 21 and 20 as a reference.

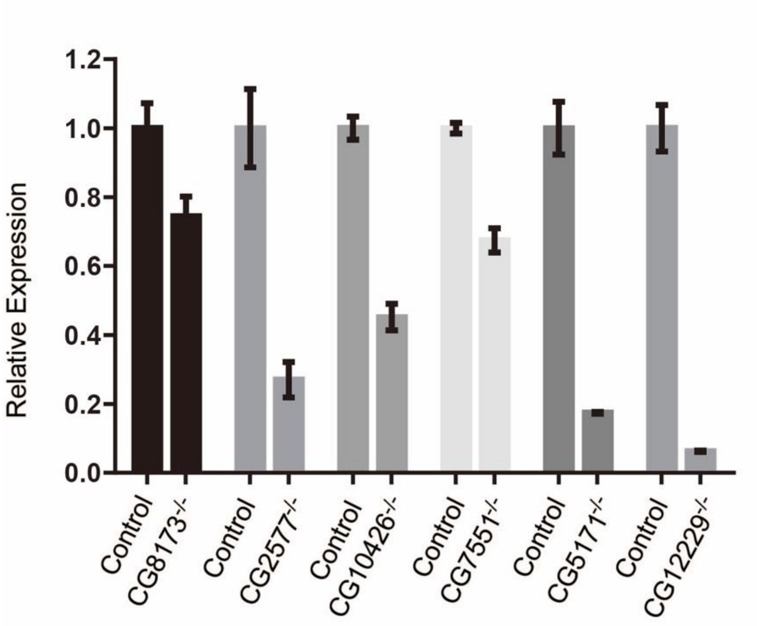

Supplementary Figure 2. Random qRT-PCR verification of our resource. CG8173, CG2577, CG10426, CG7551, CG5171 and CG12229 mRNA levels were quantified by qRT-PCR for control, CG8173$^{-/-}$, CG2577$^{-/-}$, CG10426$^{-/-}$, CG7551$^{-/-}$, CG5171$^{-/-}$ and CG12229$^{-/-}$, respectively. Error bars represent the means ± SD values of three independent experiments.

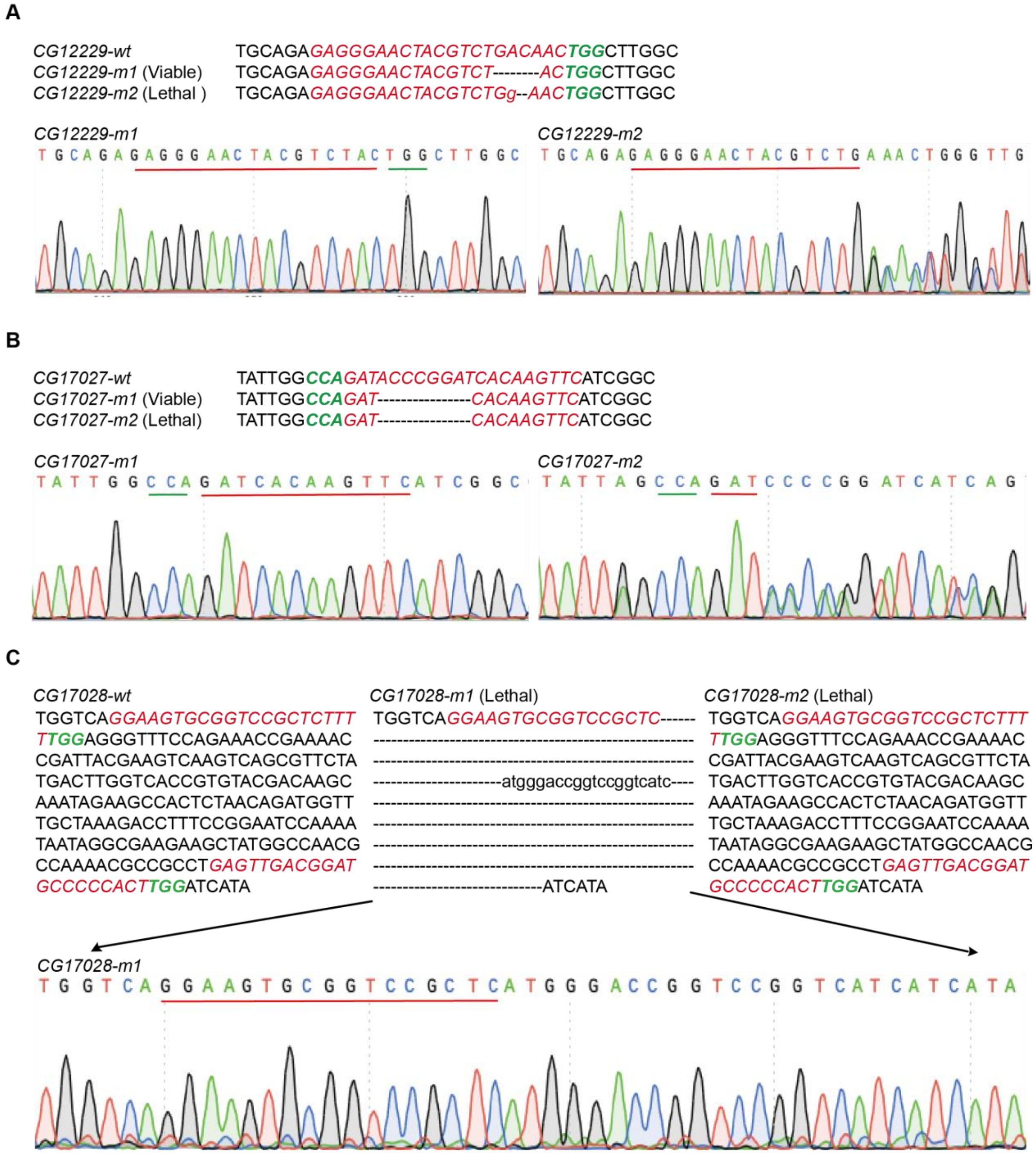

Supplementary Figure 3. Abnormal lethal events in the CRISPR/Cas9 resource. (A, B) Example frameshift viable and lethal allele sequences for the same gene. Red area, predicted gRNA recognition site. Green area, PAM sequence. (C) Large deletion of *CG17028*-m1 sequenced by gel purification of the lower PCR product from heterozygous F2. All DNA sequences were obtained from the flybase website (flybase.org) and the alignments were performed by CLUSTALW.

Supplementary Note 1: Targeted indel mutations mediated by Cas9/gRNA at 385 mutant alleles for 105 genes.

Note: wt, wild-type DNA sequence; m#, mutation line# DNA sequence. Deletions are shown as red dashes and insertions as highlighted in blue and lowercase letters. The change of DNA length (in nucleotides) caused by each mutation is indicated as purple (+, insertion; -, deletion). Note that some alterations have both insertions and deletions of nucleotides and in these cases the alterations are enumerated in the brackets. All DNA sequences were obtained from the flybase website (flybase.org) and the alignments were performed by CLUSTALW.

CG10702-wt
GGCAACATGCTGCTGGGCGTTGGCCTCATGTTCCTGGCGCTATATGTCCCCTGCGCCGAA……ATGTTCCC
GCACCTGACCGTC
CG10702-m1
GGCAACATGCTGCTGGGCGTTGGCCTCATG------------------------------……---------CACCTGACCGTC **[-258]**
CG10702-m2
GGCAACATGCTGCTGGGCGTTGGCCTCATG------------------------------……---------CACCTGACCGTC **[-258]**
CG10702-m3
GGCAACATG----------------aCATaTaTaTGGCGCTATATGTCCCCTGCGCCGAA……ATGTTCCCGCACCTGACC
GTC **[-20,+4]**

CG11221-wt
TCGATCCTTCGGCGACGGTGGTAGCACCAAC……AGCGTGAGCCGCCGGAGCAGCATCTACAAGAAGCCG
GACAAGAATGACGGCGGACAGATCCATCTCATTCCGGACGTGGAGCTGCCGCTGATGACCTT
CG11221-m1
TCGATCCTTCGGCGACGGTGGTAGCACCAAC……AGCGTGAGCCGCCGGAGCAGCATCTACAAGAAGCCG
GACAAGAATGACGGCGGACAG---------ATTCCGGATGTGGAGCTGCCGCTGATGACCTT **[-9]**
CG11221-m2
TCGATCCTTCGG------TGGTAGCACCAAC……AGCGTGAGCCGCCGGAGCAGCATCTACAAGAAGCCGGACA
AGAATGACGGCGGACAGATCCATCTCATTCCGGACGTGGAGCTGCCGCTGATGACCTT **[-6]**
CG11221-m4
TCGATCCTTCG---ACGGTGGTAGCACCAAC……AGCGTGAGCCGCCGGAGCAGCATCTACAAGAAGCCGGA
CAAGAATGACGGCGGACAGATCCATCT----CCGGATGTGGAGCTGCCGCTGATGACCTT **[-7]**
CG11221-m6
TCGATCCTTCGGCGACGGTGGTAGCACCAAC……AGCGTGAGCCGCCGGAGCAGCATCTACAAGAAGCCG
GACAAGAATGACGGCGGACAGATCCtTCg-ATTCCGGACGTGGAGCTGCCGCTGATGACCTT **[-3,+2]**

CG14006-wt
AGCCCCACATCCAGGAAGGACAGACGCTGCTTAACATGCTGTACGTGAACAACCAGGTCC……TGCGCCTG
CAGATAGCCGCTGGCGAAGATTACACTCAGTATAAAGGAGCCACCGTGCG
CG14006-m1
AGCCCCAC-----GGAAGGACAGACGCTGCTTAACATGCTGTACGTGAACAACCAGGTCC……TGCGCCTGCA
GATAGCCGCTGGCGAAGATTACACTCAGTATAAAGGAGCCACCGTGCG **[-5]**
CG14006-m2
AGCCCCACATC---GAAGGACAGACGCTGCTTAACATGCTGTACGTGAACAACCAGGTCC……TGCGCCTGCA
GATAGCCGCTGGCGAAGATTACACTCAGTATAAAGGAGCCACCGTGCG **[-3]**
CG14006-m3
AGCCCCACATCCAG-----ACAGACGCTGCTTAACATGCTGTACGTGAACAACCAGGTCC……TGCGCCTGCAG
ATAGCCGCTGGCGAAGATTACACTCAGTATAAAGGAGCCACCGTGCG **[-5]**
CG14006-m4
AGCCCCACAT---GGAAGGACAGACGCTGCTTAACATGCTGTACGTGAACAACCAGGTCC……TGCGCCTGCA
GATAGCCGCTGGCGAAGATTACACTCAGTATAAAGGAGCCACCGTGCG **[-3]**
CG14006-m5
AGCCCCACATCCAGGAAGGACAGACGCTGCTTAACATGCTGTACGTGAACAACCAGGTCC……TGCGCCTG
CAGATAGCCGCTGGCGAAGATTACACTCAcTATAtAtcGGAGCCACCGTGCG **[+4,-2]**

CG17010-wt
CCGGCTTACGCAGCCCTTTCAAATGGGAAAGAAGCTGGATGTGGTTGTCTTCGGGACGGCAAACATCGAAT
ATAT
CG17010-m1

CCGGCTTACGCAGCCCTTTCAAATGGGAAAGAAGCTGGATGTGGTTG---TCGGGACGGCAAACATCGAATAT
AT **[-3]**
CG17010-m2
CCGGCTTACGCAGCCCTTTCAAATGGGAAAGAAGCTGGATGTGGTTGTCTT--------CAAACATCGAATATAT **[-8]**
CG17010-m3
CCGGCTTACGCAGCCCTTTCAAATGGGAAAGAAGCTGGATGTGGTTGTCTTCGG-ACGGCAAACATCGAATA
TAT **[-1]**

CG2964-wt
CATGTCGTTGTCCGCGAAATCCGTGCTAAAGGAGGGCTCCACACAGCTGAGCCACATCTG……CAACGAGG
CGCTGGAGAGGATTCATAAGGAGACGGGCCAAATCCGAACGGTCGCCATTGC
CG2964-m1
CATGT---……--------------------------------CGGGCCAAATCCGAACGGTCGC
CATTGC **[-267]**
CG2964-m2
CATGTCGTTGTCCGCGAAATCCGTGCTAAAGGAGGGCTCCACACAGCTGAGCCACATCTG……CAACGAGG
CGCTGGAGAGGATTCATAAGA---CGGGCCAAATCCGAACGGTCGCCATTGC **[-3]**
CG2964-m3
CATGTCGTTGTCCGCGAAATCCGTGCTAAAGGAGGGCTCCACACAGCTGAGCCACATCTG……CAACGAGG
CGCTGGAGAGGATTCATA------CGGGCCAAATCCGAACGGTCGCCATTGC **[-6]**
CG2964-m4
CATGTCGTTGTCCGCGAAATCCGTGCTAAAGGAGGGCTCCACACAGCTGAGCCACATCTG……CAACGAGG
CGCTGGAGAGGATTCATAA-GAGACGGGCCAAATCCGAACGGTCGCCATTGC **[-1]**

CG31714-wt
GCTGCTGACGCTCCTGCTGCCGGCGGTCCTGCCACACCAGACGCCTCCAGATGCAGACCA……CTCCTATT
CCTCGTCCAGTCCAGGATGCGGGGGTATGCTAAAGGCACGCCATGGCAACAT
CG31714-m1
GCTGCTGACGCTCCTGCTGCCGGCGGTCCTGCCACACCAGACGCCTCCAGATGCAGACCA……CTCCTATT
CCTCGTCCAGTCCAGGATGCGGGGGTATGCTAAAGG--CGCCATGGCAACAT **[-2]**
CG31714-m2
GCTGCTGACGCTCCTGCTGCCGGCGGTCCTGCCACACCA------------------------……-------------------------------------
------------GGCAACAT **[-193]**
CG31714-m3
GCTGCTGACGCTCCTGCTGCCGGCGGTCCTGCCACACCAGACGCCTCCAGATGCAGACCA……CTCCTATT
CCTCGTCCAGTCCAGGATGC---------------------CATGGCAACAT **[-21]**
CG31714-m4
GCTGCTGACGCTCCTGCTGCCGGCGGTCCTGCCACACCAGACGCCTCCAGATGCAGACCA……CTCCTATT
CCTCGTCCAGTCCAGGATGCGGGGGTATG---------CaCCATGGCAACAT **[-10,+1]**
CG31714-m5
GCTGCTGACGCTCCTGCTGCCGGCGGTCCTGCCACACCAGACGCCTCCAGATGCAGACCA……CTCCTATT
CCTCGTCCAGTCCAGGATGCGGGGGTATGCaTAcAGG---ATGttGCAACAT **[-7,+4]**

CG3277-wt
GAAATCACAGAGCTGACCACAAATGTCACGCTCTACATGGAGAAAATACCACGCCTACAGGTGCGA
CG3277-m1
GAAATCACAGAGCTGACCACAAATGTCACGCTCTACATGGAGAAAATACCACG--------TGCGA **[-8]**

CG34380-wt
ACCACGCCCACAGCAGCCGCTCCCCGCCCTCCATGCCGCGCCCCCCGCTGGACGCTGTGCCTCTGGACG
CTGGTGATCGCCAGCGGCTGTTTAATGCTCAACGCACCGACGACGATGGCA
CG34380-m1
ACCACGCCCACAGCAGCCGCTCCCCGCCCTCCATGCCGCGCCCCCCGCTGGACGCTGTGCCTCTGGACG
CTGGTGATCGC----GGCTGTTTAATGCTCAACGTACCGACGACGATGGCA **[-4]**
CG34380-m2
ACCACGCCCACAGCAGCCGCTCCCCGCCCTCCATGCCGCGCCCCCCGCTGGACGCTGTGCC-------------------
-AGCGGCTGTTTAATGCTCAACGTACCGACGACGATGGCA **[-20]**

CG3544-wt
TGGACATGCTGATGGACTGTTTAGTGAAACAGGGAGCGGATATGCACACGGTGGTCTCTATTGCCGGAGCT
GCTCAGCAGCACGGTTGCGTC……CGGACGTGCAGGTCCGTGAAATGGAGCACACCGTCG
CG3544-m1

TGGACATGCTGATGGACTGTTTAGTGAAACAGGGAGCGGATATGC---CGGTGGTCTCTATTGCCGGAGCTG
CTCAGCAGCACGGTTGCGTC……CGGACGTGCAGGTCCGTGAAATGGAGCACACCGTCG **[-3]**

CG3544-m2
TGGACATGCTGATGGACTGTTTAGTGAAACAGGGAGCGGATATGCACAaCGGTGGTCTCTATTGCCGGAGC
TGCTCAGCAGCACGGTTGCGTC……CGGACGTGCAGGTCCGTGAAATGGAGCACACCGTCG **[+1]**

CG3544-m3
TGGACATGCTGATGGACTGTTTAGTGAAACAGGGAGCGGATAT--……---------
--GCAGGTCCGTGAAATGGAGCACACCGTCG **[-177]**

CG3544-m4
TGGACAT----AT---C------------C-GG-------------CACGGTGGTCTCTATTGCCGGAGCTGCTCAGCAGCACGGTTG
CGTC……CGGACGTGCAGGTCCGTGAAATGGAGCACACCGTCG **[-33]**

CG3544-m5
TGGACATGCTGATGGACTGTTTAGTGAAACAGGGAGCGGATATGCACAtCtaTGGTCTCTATTGCCGGAGCTG
CTCAGCAGCACGGTTGCGTC……CGGACGTGCAGGTCCGTGAAATGGAGCACACCGTCG **[+3,-2]**

CG4629-wt
CGACGACGACGCCCCTGACAATTTGTCAGCCGCCACCGCCCACTCCGCCCTACCAGCGGCTCACCAAGGC
GCTGCAATGCGATCCGCGCTGCGGCCACGAGGTGAGTGCAAATTGAA

CG4629-m1
CGACGACGACGCCCCTGACAATTTGTCAGCCGtgggAGCCCcccACTCCGCCCTACCAGCGGCTCACCAAGG
CGCTGCAATGCGATCCGCGCTGCGGCCACGAGGTGAGTGCAAATTGAA **[+7,-4]**

CG4629-m2
CGACGACGACGCCCCTGACAATTTGTCAGCCGCCACC----ACTCCGCCCTACCAGCGGCTCACCAAG--GCT
GCAATGCGATCCGCGCTGCGGCCACGAGGTGAGTGCAAATTGAA **[-6]**

CG4629-m3
CGACGACGACGCCCCTGACAATTTGTCAGCCGtgggAGCCCcccACTCCGCCCTACCAGCGGCTCACCAAG-G
GCTGCAATGCGATCCGCGCTGCGGCCACGAGGTGAGTGCAAATTGAA **[+7,-5]**

CG4629-m4
CGACGACGACGCCCCTGACAATTTGTCAGCCGCCAC-------TCCGCCCTACCAGCGGCTCACCAAGGGC-TG
CAATGCGATCCGCGCTGCGGCCACGAGGTGAGTGCAAATTGAA **[-8]**

CG4839-wt
GCCGAAAATGGCAGCGCAAACAATGGCGTCCAGGAGCGGAAGTTCCGCACCAA……AGCGGGTGCAGCCG
GGTGC

CG4839-m1
GCCGAAAA------------CAATGGCGTCCAGGAGAGGAAGTTCCGCACCAA……AGCGGGTGgcgctgCAGCCGGG
TGC **[-12,+6]**

CG4839-m2
GCCGAAAATGGCA---------------TCCAGGAGAGGAAGTTCCGCACCAA……AGCGGGTGgcgctgCAGCCGGGTG
C **[-15,+6]**

CG5790-wt
AAAGTCGGCGACCAACATGGATTGTGCTAATACGAATTTAGAATCGGCAGGGAAGA……CCGGAACTATGC
CTTCCGTTTCGGGCACTAACCATCATTCTAGGCATCAACAGCTCCTTC

CG5790-m1
AAAGTCGGCGAC---CATGGATTGTGCTAATACGAATTTAGAATCGGCAGGGAAGA……CCGGAACTATGCCT
TCCGTTTCGGGCACTAACCATCATTCTAGGCATCAACAGCTCCTTC **[-3]**

CG5790-m2
AAAGTCGGCGACtAtCtgACATGGATTGTGCTAATACGAATTTAGAATCGGCAGGGAAGA……CCGGAACTATG
CCTTCCG------GCACTAACCATCATTCTAGGCATCAACAACTCCTTC **[+4,-6]**

CG5790-m3
AAAGTCGGCGACtttAgCACATGGATTGTGCTAATACGAATTTAGAATCGGCAGGGAAGA……CCGGAACTATG
CCTTCCG-----GGCACTAACCATCATTCTAGGCATCAACAACTCCTTC **[+4,-5]**

CG5790-m4
AAAGTCGGCGACttAgCATGGATTGTGCTAATACGAATTTAGAATCGGCAGGGAAGAGTT……GAACTATGCC
T--------aGGC--------ATAGACTAGGCATCAACAACTCCTTC **[+4,-19]**

CG5790-m5
AAAGTCGGC---tAAtAcGGAcTGTGCTAATACGAATTTAGAATCGGCAGGGAAGA……CCGGAACTATGCCTTC
CGTTTCGGGCACTAACCATCATTCTAGGCATCAACAGCTCCTTC **[-7,+4]**

CG7094-wt
GCCCATTGGGTCCGGGTCCTTCGGCGATATATATCTCGGACTAAGCATCACGGATGGATCCGAGGTGGCCA
TTAAGGTGGAGAAGAACGATGCCAAGTACCCGCAGCTGATATACGAGGC

CG7094-m1
GCCCATTGGGTCCGGGTCCTTCGGCGATATATATCTCGGACTAAGCATCACGGATGGATCCGAGGcaaGCAT
------------------GCCAAGTACCCGCAGCTGATATACGAGGC **[-22,+3]**

CG7094-m2

GCCCATTGGGTCCGGGTCCTTCGGC--T------CTC--tCT-----TC----------T-CC--------TTAAGGTGGAGAAGAACG
ATGCCAAGTACCCGCAGCTGATATACGAGGC **[-35,+1]**

CG7094-m3

GCCCATTGGGTCCGGGTCCTTCGGCGATATATATCTCGGACTAAGCATCACGGATGGATCCGA------CATTAA
GGTGGAGAAGAACGATGCCAAGTACCCGCAGCTGATATACGAGGC **[-6]**

CG7094-m4

GCCCATTGGGTCCGGGTCCTTCGGCGATATATATCTCGGACTAAGCATCACGGATGGATCCGAGGTG---------
GTGGCGAAGAACGATGCCAAGTACCCGCAGCTGATATACGAGGC **[-9]**

CG7094-m5

GCCCATTGGGTCCGGGTCCTTCGGCGATATATATCTCGGACTAAGCATCACGGATGGATCCGAGGTG---------
---GAGAAGAACGATGCCAAGTACCCGCAGCTGATATACGAGGC **[-12]**

CG7094-m6

GCCCATTGGGTCCGGGTCCTTCGGCGATATATATCTCGGACTAAGCATCACGGATGGATCC-A-----CC-TTAA
GGTGGAGAAGAACGATGCCAAGTACCCGCAGCTGATATACGAGGC **[-7]**

CG7236-wt

TCAGTCGGCTGGGCGAGGGCTCCTACGGTGTGGTCTACAAGTGCCGGGATCGGGAAACGG

CG7236-m1

TCAGTCGGCTGGGCGA---CT-----GG----G-CTACAgGTGCCGGGATCGGGAAACGG **[-13]**

CG7236-m2

TCAGTCGGCTGG---------------GTGTGGTCTACAAGTGCCGGGATCGGGAAACGG **[-15]**

CG7236-m3

TCAGTCGGCTGGGCGAGGGCTC----GGTGTGGTCTACAAGTGCCGGGATCGGGAAACGG **[-4]**

CG9222-wt

GTGGTACCCTTTTTAATGGCGACATCTCCCAGAAGAAAAAACGATCAAGGCGGAGG……ACTCGGGTCGTC
GTCAGGAGCAGAAGGTCTACACCTTCAGTGACCGGCCACCACAACCAAAGCCACCGGCACCATCCGGA

CG9222-m1

GTGGTACCCTTTTTAATGGCGACATCTCCCAGAAGAAAAAACGATCA-GGCGGAGG……ACTCGGGTCGTCG
TCAGGAGCAGAAGGTCTACACCTTCAGTGACCGGCCACCACAACCAAAGCCACCGGCACCATCCGGA **[-1]**

CG9222-m2

GTGGTACCCTTTTTAATGGCGACATCTCCCAGAAGAAAAAACGATCAAGGCGGAGG……ACTCGGGTCGTC-
----------------TACACCTTCAGTGACCGGCCACCACAACCAAAGCCACCGGCACCATCCGGA **[-17]**

CG9541-wt

TTTGTTTTAGGCGGCCCCGGTAGTGGCAAGGTCACCCATTGCGATACCTTCATGCAGGAG

CG9541-m1

TTTGTTTTAG-------------TGGCAAGGTCACCCATTGCGATACCTTCATGCAGGAG **[-13]**

CG9541-m2

TTTGTTTTAGGCGGCCCCGGT-GTGGCAAGGTCACCCATTGCGATACCTTCATGCAGGAG **[-1]**

CG10376-wt

GGTGGTCAGCCGGAGTTGTGTAACTCGTACGGCCAATGAGACATACAAAGTGTCCGGCGAGGAGCGTCAT
GCCGAATTGGTCTCAGCTATTTG

CG10376-m1

GGTGGTCAGCCGGAGTTGTGTAACTCGTACGGCCAtTGtAtgtacgGACATACAAAGTGTCCGGCGAGGAGCGT
CATGCCGAATTGGTCTCAGCTATTTG **[+7]**

CG10376-m2

GGTGGTCAGCCGGAGTTGTGTAACTCGTACGGCCAtTGGcCgTACATACAAAGTGTCCGGCGAGGAGCGTCA
TGCCGAATTGGTCTCAGCTATTTG **[+3]**

CG10376-m3

GGTGGTCAGCCGGAGTTGTGTAACTCGTACGGCCA-------------------------GGAGCGTCATGCCGAATTGGTCTC
AGCTATTTG **[-25]**

CG10376-m4

GGTGGTCAGCCGGAGTTGTGTAACTCGTACGGC--------CATACAAAGTGTCCGGCGAGGAGCGTCATGCCG
AATTGGTCTCAGCTATTTG **[-8]**

CG15385-wt

TCGCCAAGTACAGAGAACGCTGTGCTCCGTTGGCTCAGCTGCAGCGTTTGGATGATGGCGGCATCTTGGAG
GGTTGGAAGCTTCAGGGCGTGCTCCTGGTCATCAGGCACGGGGATCGGG

CG15385-m1

TCGCCAAGTACAGAGAACGCTGTGCTCCGTTGGCTCAGCTGCAGCGTTTGGA------------------GGGTTGGAAG
CTTCAGGGCGTGCTCCTGGTCATCAGGCACGGGGATCGGG **[-18]**

CG15385—m2

TCGCCAAGTACAGAGAACGCTGTGCTCCGTTGGCTCAGCTGCAGCGTTTGGATGATGGCGG----TTGGAGG
GTTGGAAGCTTCAGGGCGTGCTCCTGGTCATCAGGCACGGGGATCGGG **[-4]**

CG15385-m3
TCGCCAAGTACAGAGAACGCTGTGCTCCGTTGGCTCAGCTGCAGCGTTTGGATGATGGCG-----------GGTTG
GAAGCTTCAGGGCGTGCTCCTGGTCATCAGGCACGGGGATCGGG **[-11]**

CG16771-wt
GTTTCGAAAACTCAGCGATGTGGACATGACGGAAGTGCAGCTGAACAATTTGGATGACTC
CG16771-m1
GTTTCGAAAACTCAGCGATGTGGACATGACGGAAGTGCAGCTG---AATTTGGATGACTC **[-3]**
CG16771-m2
GTTTCGAAAACTCAGCGATGTGGACATGACGGAAGTGCAGCTGA--AATTTGGATGACTC **[-2]**

CG17124-wt
AACGAGAAGGGGGCGGAGGTGAAGGAGCGGCGGGAGAAGTTCCTCACGGCCAAGTACGGT
CG17124-m1
AACG-G-------------------------------------T-ACG-----GTACGGT **[-44]**
CG17124-m2
AACGAG--GGGGGCGGAGGTGAAGGAGCGGCGGGAGAAGTTCCTCACGGCCAAGTACGGT **[-2]**
CG17124-m3
AACG--GT **[-54]**
CG17124-m4
AACGA---GGGGGCGGAGGTGAAGGAGCGGCGGGAGAAGTT------G-----GTACGGT **[-14]**
CG17124-m5
AACGAGgtGGGG-CGGAGGTGAAGGAGCGGCGGGAGAAGTTCCTCACGGCCAAGTACGGT **[-3,+2]**

CG5171-wt
ACCTGTAATTAGTAATCTCGAGGATTTTGCGAATAATTTACCTGGGTAAGTCGGAGTTAT……AGCGAAGACC
AAAATGCCCGTGGAACTGGAGGCCATACTGCACAAGATAGCCAAGCATCCCAAAGT
CG5171—m1
ACCTGTAATTAGTAATCTCGAGGATTTTGCGAATAATTTACCTGGGTAAGTCGGAGTTAT……AGCGAAGACC
AAAATGCCCGTGGAACTGGAGGCCATACTGCACAAGATAGCCAAGCA-CCCAAAGT **[-1]**
CG5171-m2
ACCTGTAATTAGTAATCTCGAGGATTTTGCGAATAA-------------GTCGGAGTTAT……AGCGAAGACCAAAATG
CCCGTGGAACTGGAGGCCATACTGCACAAGATAGCCAAGCATCCCAAAGT **[-13]**
CG5171-m3
ACCTGTAATTAGTAATCTCGAGGATTTTGCGAATAATTTACCTGGGTAAGTCGGAGTTAT……AGCGAAGACC
AAAATGCCCGTGGAACTGGAGGCCATACTGCACAAGATAGCC--------CAAAGT **[-8]**
CG5171—m4
ACCTGTAATTAGTAATCTCGAGGATTTTGCGAATAATTTACCTGGGTAAGTCGGAGTTAT……AGCGAAGACC
AAAATGCCCGTGGAACTGGAGGCCATACTGCACAAGATAGCCAAGCAaTCCCAAAGT **[+1]**

CG5177-wt
AGAAGGCACCATTGCTGAAGAAGGAGGAGGACTACGTGAAGGCCCTCGAGGGGTGAGTTT……GCTCACC
GAGGAGCTGTCCGTCATGCCCAAGGATACCGAGATCAACATCAAGAAACTGGC
CG5177-m1
AGAAGGCACCA---……-------------------------------AGGATACCGAGATCAACATC
AAGAAACTGGC **[-198]**

CG6380-wt
CACATGATCATCGACGAACCAAAGACACCCTTCGTCTTTGAGGAGGATTTGCCAAAGGAGTTGGATACAAAT
GCCCTGATCGAAAAGTTGAGACACACATCAAAGTCAGAAATGCCGGCA
CG6380-m1
CACATGATCATCGACGAACCAAAGACACCCTTCG-----------------------------GATACAAATGCCCTGATCGAAAAG
TTGAGACACACATCAAAGTCAGAAATGCCGGCA **[-29]**
CG6380-m2
CACATGATCATCGACGAACCAAAGACACCCTTCGTCTTTGAAGAGGATTTGCCAAAG---TTGGATACAAATGC
CCTGATCGAAAAGTTGAGACACACATCAAAGTCAGAAATGCCGGCA **[-3]**
CG6380-m3
CACATGATCATCGACGAACCAAAGACACCCTTCGTCTTTGAAGAGGATTTGCCAAAGGAtacaaaGTTGGATAC
AAATGCCCTGATCGAAAAGTTGAGACACACATCAAAGTCAGAAATGCCGGCA **[+6]**

CG7115-wt
CTCTATTTGCAAGCGGTAGATGTGTGGAGTCGCAGCATCCTGGGACGCATCCAGGCAACCCTCGGTCGCCA
GAAG
CG7115-m1
CTCTATTTGCAAGCGGTAGATGTGTGGAGTCGCAGCATCCTGGGACGCATCC---------TgGGTCGCCAGAAG
[-10,+1]
CG7115-m2

CTCTATTTGCAAGCGGTAGATGTGTGGAGTCGCAGCATCCTGttttaGaGctAgaaatagCaagttaaaataagGCtAgTC
CgttAtcaacttgaaaaagtGGCACCGaGTCGCCAGAAG **[+45]**
CG7115-m3
CTCTATTTGCAAGCGGTAGATGTGTGGAGTCGCAGCATCCTGGG---------------CCTCGGTCGCCAGAAG **[-15]**
CG7115-m4
CTCTATTTGCAAGCGGTAGATGTGTGGAGTCGCAGCA------GA---A----GG----CCTCGGTCGCCAGAAG **[-17]**

CG7180-wt
GCAGAGGAAAAGCCGAGCCATCGTCTCCATACCGAACACCATCAAACTGAGTATGCTCAA
CG7180-m1
GCAGAGGAAAAGCCGAGCCATC-----CATACCGAACACCATCAAACTGAGTATGCTCAA **[-5]**

CG10082-wt TTTCTTGGGGGATTGGGGCATGGGAGATTCTGACTTGTCGTCATATCAGCAACATGTTC
CAAATATACAACAGCCGCGAC……CCACAGCAGCAGCAGCAGCAGCAGC……GTTGCAGTTCGCCAGTGGA
TCCAGCTTCCTGCAACAAGGCTCAT
CG10082-m1
TTTCTTGGGGGATTGGGGCATGGGAGATTCTGACTTGTCGTCATATCAGCAACATGTTCCAAATATACAACA
GCCGCGAC……CCACAGCAGCAG------CAGCAaC……GTTGCAGTTCGCCAGTGGATCCAGCTTCCTGCAAC
AAGGCTCAT **[-7,+1]**
CG10082-m2
TTTCTTGGGG----G------------------------……--
--------ACAAGGCTCAT **[-244]**
CG10082-m3
TTTCTTGGGGG----CACCGACTCGGTGCCACTTTTTCAAGTTGATAACGGACTAGCCTTATTTTAACTTGCTAT
TTCTAGCTCTAAAACAATCCCCCAAGAAATATACCTATAGTGGTCGTATTAAGG----……--CTCGGACAAGGCT
CAT **[-125]**
CG10082-m4
T------------------------AACAAGG------……---
-----------CTCAT **[-253]**

CG10459-wt
TGTGGTCGGTATCCTTAAGCCACGATGCCAAGATCCTGGCCAGTGGCGGTGCAGACTGCA
CG10459-m1
TGTGGTCGGTATCC---------GATGCCAAGATCCTGGCCAGTGGCGGTGCAGACTGCA **[-9]**
CG10459-m2
TGTGGTCGGTATCC-----------TGCCAAGATCCTGGCCAGTGGCGGTGCAGACTGCA **[-11]**

CG1344-wt
ACTAAAAGGAATACTTGTTGAGGAAAATGCGGTGGAGACTAATCAATTTTGGACAGTT……TCAGACCTCTAG
ATGAGGTGCTGGCCCAGCAGACTGACATTGAAGTCTGCCTAGG
CG1344-m1
ACTAAAAGGAATACTTGTTGAG------------------------------------……--------------------------------ACTGACATTGAAGTC
TGCCTAGG **[-340]**
CG1344-m2
ACTAAAAGGAATACTTGTTGAGGAAAATGCGGTGGAGACTAATCAATTTTGGACAGTT……TCAGACCTCT----
---------------------GACATTGAAGTCTGCCTAGG **[-25]**
CG1344-m3
ACTAAAAGGAATACTTGTTGAGGAAtAcTTGCGGTGGAGACTAATCAATTTTGGACAGTT……TCAGACCTCTA
GATGAGGTGCTGGCCCAGCAGACTGACATTGAAGTCTGCCTAGG **[+2]**

CG17528-wt
AGGCTTCTATTATCGAAAAGACGAATCAACCACATAATGTTCAGGAAGATAATAACTACA
CG17528-m1
AGGCTTCTATTATCGAAAAGACGAATCAACCACATAtAaT------AGATAATAACTACA **[-8,+2]**

CG3216-wt
GGGCTCCTGGACTGGCTGCGGACATGTACTTCCAGGACGACGTAATCGCGTTTATTGGAC
CG3216-m1
GGGCTCCTG---------CGGACATGTACTTCCAGGACGACGTAATCGCGTTTATTGGAC **[-9]**
CG3216-m2
GGGCTCCTG-----GCTGCGGACATGTACTTCCAGGACGACGTAATCGCGTTTATTGGAC **[-5]**

CG33671-wt
GCTGGAGGACAGCCAGGTGGCAAGTTTTCAACTGGAGGCCCTTAACTGCACACT
CG33671-m1
GCTGGAGGACAGCCAGGTGGCAAG-------------GCCCTTAACTGCACACT **[-13]**

CG33671-m2
GCTGGAGGACAGCCAGGTGGCAAGTTTcCAcCTGGAGGtggagGCCCTTAACTGCACACT **[+7,-1]**

CG3494-wt
GTGACGGATTGAACAGCCCCAGGCTGCAGGGAGGAAAGGAAGCGGAAGCGGATGGGAGTC……CTCAAAT
CAGCGAGGTGCCCATGGAGGTATTCGAGGCAGCTCAGCAGGAGCTAGTGAA
CG3494-m1
GTGACGGATTGAACAGCCCTAGGCTGCAGGGAGGAAAGGAAGCGGAAGCAGATGGGAGTC……CTCAgAT
CA---AG--GCtC----AG--A-TCGAGGCAGCTCAGCAGGAGCTAGTGAA **[-14,+2]**
CG3499-m2
GTGACGGATTGAACAGCCCCAGGCTGCAGGGAGGAAAG------GAAGCGGATGGGAGTC……CTCAAATCAG
CGAGGTGCCCATGGAGGTATTCGAGGCAGCTCAGCAGGAGCTAGTGAA **[-6]**
CG3494-m3
GTGACGGATTGAACAGCCCCAGGCTGCAGGGAGGAAAG------GAAGCGGATGGGAGTC……CTCAGATCAG
CGAGGTGCCCATGGAGGTAgcTTCGAGGCAGCTCAGCAGGAGCTAGTGAA **[-6,+2]**

CG3608-wt
TTTGTGCAGACCATGAAGGTACTTCACTCCGATGCGCCGCAAAACCCCATTGAGGACCTG
CG3608-m1
TTTGTGCAGAC----AAc--ACTTCACTCCGATGCGCCGCAAAACCCCATTGAGGACCTG **[-7,+1]**
CG3608-m2
TTTGTGCAGA------AGGTACTTCACTCCGATGCGCCGCAAAACCCCATTGAGGACCTG **[-6]**

CG4945-wt
CTACTGGTGGAGCATCGGGGATCACAGACGGAGATGGTCCTGAAAGCTGTACCCAAACCA……CAGGAGTA
TGCTCCACTAGGTGGGTAACTCCTATAATAACAATATTTATATATCTATATA
CG4945-m1
CTACTGGTGGAGCATCGGG-------------GATGGTCCTGAAAGCTGTACCCAAACCA……CAGGAGTATGCTCCA
CTAGGTGGGTA--TCCTATACTAACAATATTTATATATCTATATA **[-15]**
CG4945-m2
CTACTGGTGGAGCATCGGGGATCACAGACGGAGATGGTCCTGAAAGCTGTACCCAAACCA……CAGGAGTA
TGCTCCACTAGGTGGG--ACTCCTATAATAACAATATTTATATATCTATATA **[-2]**
CG4945-m3
CTACTGGTGGAGCATCGGG-------ACGGGAGATGGTCCTGAAAGCTGTACCCAAACCA……CAGGAGTATGC
TCCACTAGGTGGGTA--TCCTATACTAACAATATTTATATATCTATATA **[-9]**

CG5757-wt
GCGCGGGAAAACGTGGAGCGTTGATAATATTTGAAGGATGTGATCGAAGTGGAAAGACCA
CG5757-m1
GCGCGGGAAAACGTGGAGCGTTGATAATATTgTGAAGGATGTGATCGAAGTGGAAAGACCA **[+1]**

CG8179-wt
ATCGGGAAAGGATGGCCGGCGCAAGGTCACGGAGTGCGGATCAGTGCGACGGAGCAGTGG
CG8179-m1
ATCGGGAAAGGAT------CGCAAGGTCACGGAGTGCGGATCAGTGCGACGGAGCGGTGG **[-6]**

CG8726-wt
TATGCTCTGCCTGCGCAACGACACTACGTGGTCGCTGGGTGGAAGTATGGGTGCCATAGGTATGCT
CG8726-m1
TATGCTCTGCCTGCGCAACGACACTACGTGGTCGCTGGGTGGAAGTATGGGTGCCA--GGTATGCT **[-2]**
CG8726—m2
TATGCTCTGCCTGCGCAACGACACTACGTGGTCGCTGGGTGGAAGTATGGGTGCCAT-GGTATGCT **[-1]**

CG8878-wt
ACGCAAGCGAAAGCGTTCTGCGGTCAAGGCCGCCGAGAAGCGCCAGCGCTTGAGTGGTGGTTCCAGCAGC
GCCAATGGCTTC
CG8878-m1
ACGCAAGCGAAAGCGTTCTGCGGTCAAGGCCGCCGAGAAGCGCCAGCG--------------------CGCCAATGGCTT
C **[-20]**
CG8878-m2
ACGCAAGCGAAAGCGTTCTGCG----------------------------------GTGGTTCCAGCAGCGCCAATGGCTTC **[-34]**
CG8878-m3
ACGCAAGCGA--------TG-G-------CC-------------------TG-Ga--TGGTTCCAGCAGCGCCAATGGCTTC **[-39,+1]**
CG8878-m4
ACGCAAGCGAAAGCGTTCTGCGGTCAAGGCCGCCGAGAAGCGCCAGC-C-------GTGGTTCCAGCAGCGCCA
ATGGCTTC **[-8]**

CG10417-wt
TAGCCGTTGGAGCTAGCTCTATGCAAGGATGGCGGAACAGCCAAGAGGTAAATTGAGATG
CG10417-m1
TAGCCGTTGGAGCTAGCTCTAT---AGGATGGCGGAACAGCCAAGAGGTAAATTGAGATG **[-3]**
CG10417-m2
TAGCCGTTGGAGCTAGCTCTAT----GGATGGCGGAACAGCCAAGAGGTAAATTGAGATG **[-4]**
CG10417-m3
TAGCCGTTGGAGCTAGCTCTATGC----ATGGCGGAACAGCCAAGAGGTAAATTGAGATG **[-4]**
CG10417-m4
TAGCCGTTGGAGCTAGCTCTAaGC--GGATGGCGGAACAGCCAAGAGGTAAATTGAGATG **[-3,+1]**

CG3290-wt
ACAGTCACAGCTGCTCGCATCCTGAAAGGACAACGCCAAGGAAACACTGGGGAGGAGTCC
CG3290-m1
ACAGTCACAGCTGCTCGCATCCTAAAAGGACAACGCCAA-----CACTGGGGAGGAGTCC **[-5]**

CG3292-wt
TAGCCATTAGCCTGGTGTTATGCACCCTGGTGGCCACCTCCTTGGCTGCTTCCATAG……CGCGAAGGAAA
CCTTATCGACCCAAATGCCATGGCCAAGGGAAAACCCGGACCAGAGGAGGAGAAGAA
CG3292-m1
TAGCCATTAG-CTGGTGTTATGCACCCTGGTGGCCACCTCCTTGGCTGCTTCCATAG……CGCGAAGGAAAC
CTTATCGACCCAAATGCCATGGCCAAGGGAAAACCCGGACCggtcctccAGAGGAGGAGAAGAA **[-1,+8]**
CG3292-m2
TAGCCATTAGCCTGGTGTTATGCACCCTGGTGGCCACCTCCTTGGCTGCTTCCATAG……CGCGAAGGAAA
CCTTATCGACCCAAATGCCATGGCCAAGGGAAAACCCGGACC-GAGGAGGAGAAGAA **[-1]**
CG3292-m3
TAGCCA---……---AGAAGAA **[-2
32]**
CG3292-m4
TAGCCATTAGCCTGGTGTTATGCACCCTGGTGGCCACCTCCTTGGCTGCTTCCATAG……CGCGAAGGAAA
CCTTATCGACCCAAATGCCATGGCCAAGGGAAAACCCGt--CC--GGAGGAGAAGAA **[-5,+1]**

CG3530-wt
AGCTTCCCTTGAGCACGACAGGATCTCCGCTACTCATCCGCTGCAAGACCTTCCTCTCCG……AAGGATGAT
TTCCCCAAGAACGCTGGCTGGGATTACTTTAAACTGGAGGCGGAGTTCAAG
CG3530-m1
AG-----------CACGACAGGATCTCCGCTACTCATCCGCTGCAAGACCTTCCTCTCCG……AAGGATGATTTCCC
CAAGAACGCTGGCTGGGATTACTTTAAACTGGAGGCGGAGTTCAAG **[-11]**
CG3530-m2
AGCTTCCCTTGAGCACGACAGGATCTCCGCTACTCATCCGCTGCAAGACCTTCCTCTCCG……AAGGATGAT
TTCCCCAAGAACGCTGGCTGGGATTACTTTAAACTG---GCGGAGTTCAAG **[-3]**

CG6805-wt
TGGCACCACCACTTGTCCGGATAACCAGCTGCCCGATATCTATGTAATCGGATTCCAGGAGGTGAGCACCA
CACCGCAGGTGCTAAAAATCTTCAATGACGATCCGTGGGTGCTGAAGAT
CG6805-m1
TGGCACCACCACTTGTCCGGATAACCAGCTGCCCGATATCTATGTAATCGGATTCCAGGAGGTGAGCACC---
--GCAGGTGCTAAAAATCTTCAATGACGATCCGTGGGTGCTGAAGAT **[-5]**
CG6805-m2
TGGCACCACCACTTGTCCGGATAACCAGCTGCCCGATATCTATGTAATCGGATTCCAGGAGGTGAGCACCC-
---GCAGGTGCTAAAAATCTTCAATGACGATCCGTGGGTGCTGAAGAT **[-4]**
CG6805-m3
TGGCACCACCACTTGTCCGGATAACCAGCTGCCCGATATCTATGTAATCG--------GAGGT------------GCAGGTG
CTAAAAATCTTCAATGACGATCCGTGGGTGCTGAAGAT **[-20]**

CG12484-wt
CACACGCTGGGTCCTTACAACGAGGGCTCTGGGATAAACATCACGTGCGTGGCCATCGGC
CG12484-m1
CACACGCTGGGTCCTTACAACGAGGGCTCTGGGATAAACAT----TGCGTGGCCATCGGC **[-4]**
CG12484-m2
CACACGCTGGGTCCTTACAACGAGGGCTCTGGGATAAACATCA--TGCGTGGCCATCGGC **[-2]**
CG12484-m3
CACACGCTGGGTCCTTACAACGAGGGCTCTGGGATAAACATCAtG-GCGTGGCCATCGGC **[-2,+1]**
CG12484-m4
CACACGCTGGGTCCTTACAACGAGGGCTCTGGGATAAACATCAC-TGCGTGGCCATCGGC **[-1]**

CG33672-wt
CGAATCAGACGGCTGTGGCGGCAAGTTCAGCGCCGTAATCGTATCGCCAGCCTTCAGCGGCAAGACCCTTC
TCCAGAAGCATAGATTAGTTAACTCAACGCTTGCCGAAGAACTGAAAGA
CG33672-m1
CGAATCAGACGGCTGTGGCGGCAAGTTCAGCGCCGTAATCGTATCGCCAGCCTTCA--GGCAAGACCCTTCT
CCAGAAGCATAGATTAGTTAACTCAACGCTTGCCGAAGAACTGAAAGA **[-2]**
CG33672-m2
CGAATCAGACGGCTGTGGCGGCAAGTTCAGCGCCGTAATCGTATCGCCAGCCTTCAG-GGCAAGACCCTTC
TCCAGAAGCATAGATTAGTTAACTCAACGCTTGCCGAAGAACTGAAAGA **[-1]**

CG34318-wt
CTGCAGGAGGTCGAAACCGATTCACAGGCCTGCAACGACGTGGCCAGCGTCCTT
CG34318-m1
CTGCAGGAGGTCGAAAC------ACAGGCCTGCAACGACGTGGCCAGCGTCCTT **[-6]**
CG34318-m2
CTGCAGGAGGTCGAAACCGAaaCcgaaACAGGCCTGCAACGACGTGGCCAGCGTCCTT **[+6,-2]**
CG34318-m3
CTGCAGGAGGTCGAAACCG-TTg-CAGGCCTGCAACGACGTGGCCAGCGTCCTT **[-3,+1]**

CG10738-wt
GACGACTCAGGGATCGGGGTCATTCCCTTCAGTTCGTCGTGGCCGAAACTTACG
CG10738-m1
GACGA-TCAGGGATCGGGGTCATTCCCTTCAGTTCGTCGTGGCCGAAACTTACG **[-1]**
CG10738-m2
GACGAtCcccgaTCAGGGATCGGGGTCATTCCCTTCAGTTCGTCGTGGCCGAAACTTACG **[+6]**

CG11811-wt
GGGCGCTCAGCAGCAGCAGCAGCAGTAGCAGTGCAGCGGCATCCTTAACGAGCA
CG11811-m1
GGGCGCTCAGCAGCAGCAGCAGCAGcggcagTAGCAGTGCAGCGGCATCCTTAACGAGCA **[+6]**

CG12229-wt
CTACGTCTGACAACTGGCTTGGCTCTGGAGATTAACGGCGAGTGCTGTCGCGTTGGGAGA
CG12229-m1
CTACGTCT-------GGCTTGGCTCTGGAGATTAACGGCGAGTGCTGTCGCGTTGGGAGA **[-7]**
CG12229-m2
CTACGTCT----ACTGGCTTGGCTCTGGAGATTAACGGCGAGTGCTGTCGCGTTGGGAGA **[-4]**
CG12229-m3
CTACGTCTG-gAACTGGCTTGGCTCTGGAGATTAACGGCGAGTGCTGTCGCGTTGGGAGA **[-2,+1]**

CG12289-wt
GACTACGTGACCACCATGGATAAGGTCGACTTCCAGTGCGACAGGTGCAGCACCCAGAAT
CG12289-m1
GACTACGT-------------------------------GACAGGTGCAGCACCCAGAAT **[-31]**

CG1271-wt
ATGCGAAGTCCGGGGATCGGCGGTGGATGCTGTAAGTGATTCGATCTTCAAGT
CG1271-m1
ATcGGtccAAtccGaTCtGGGGATCGGCGGTGGATGCTGTAAGTGATTCGATCTTCAAGT **[+7]**

CG34455-wt
GTTTCGATGTGGATCCACTGAACTCGGTCCAGTTCTCCAATCATACGGGCTACAAGACCT
CG34455-m1
GTTTCGATGTGGATCCACTG-----GGTCCAGTTCTCCAATCATACGGGCTACAAGACCT **[-5]**

CG7328-wt
CGTGACCATCAATGGGAGCTATCCTATGGAGGACACCGATCGGCGATGCCTGGATGGGTT
CG7328-m1
CGTGACCATCAATGGGAGCTATCCTATGGAGGACACC-------------TGGATGGGTT **[-13]**
CG7328-m2
CGTGACCATCAATGGGAGCTATCCTATGGAGGACACCGATCGGC--TGCCTGGATGGGTT **[-2]**

CG7551-wt
ATACTGGATTCGCGGTGGCAAGGCCTCGAACAACTGTACTGTTTTGGCCAATCTGGGCGT……CTTGAAGA
GTCGTGGCATCATTATCGACAACTGTCCAACCTGTGACCAGGGTGCACCCTT
CG7551-m1

ATACTGGAT---CGGTGGCAAGGCCTCGAACAACTGTACTGTTTTGGCCAATCTGGGCGT……CTTGAAGAGT
CGTGGCATCATTATCGACAACTGTCCAACCTGTGACCAGGGTGCACCCTT **[-3]**
CG7551-m2
ATACTGGATTCGCGGTGGCAAGGCCTCGAACAACTGTACTGTTTTGGCCAATCTGGGCGT……CTTGAAGA
GTCGCGGCATCATTATCGACAACTGTCCAACC----ACCAGGGTGCACCCTT **[-4]**
CG7551-m3
ATACTGGATTC-CGGTGGCAAGGCCTCGAACAACTGTACTGTTTTGGCCAATCTGGGCGT……CTTGAAGAG
TCGTGGCATCATTATCGACAACTGTCCAACCTGTGACCAGGGTGCACCCTT **[-1]**

CG7616-wt
ATGGCCTGCGAGAGGCGAATGTCCAGAAAGGCGGTCGTCCTGTATTGCGATTTAGTCTTT
CG7616-m1
ATGGCCTGCGAGAGGCGAATGTCCA---AGGCGGTCGTCCTGTATTGCGATTTAGTCTTT **[-3]**

CG10089-wt
CGCGAAGGCAACGTACTCATCCACTGCCTGGCGGGGATGTCGCGCTCGGTGACCGTGGCCGTGGCCTATA
TCATGACGGCCACACACCTGAACTGGAAGGAGGCACTTAAGGTGGT
CG10089-m1
CGCGAAGGCAACGTACTCATCCACTGCCTGGCGGGGATGTCGCGCTCG------GTGGCCGTGGCCTATATCA
TGACGGCCACACACCTGAACTGGAAGGAGGCACTTAAGGTGGT **[-6]**
CG10089-m2
CGCGAAGGCAACGTACTCATCCACTGCCTGGCGGGGATGTCGCGCTCGGT---CGTGGCCTatatcGGCCTATA
TCATGACGGCCACACACCTGAACTGGAAGGAGGCACTTAAGGTGGT **[-4,+5]**
CG10089-m3
CGCGAAGGCAACGTACTCATCCACTGCCTGGCGGGGATGTCGCGCTCGGT---------------------CATGACGGCC
ACACACCTGAACTGGAAGGAGGCACTTAAGGTGGT **[-21]**
CG10089-m4
CGCGAAGGCAACGTACTCATCCACTGCCTGGCGGGGATGTCGCGCTCGG---CCGTGGCCGTGGCCTATATC
ATGACGGCCACACACCTGAACTGGAAGGAGGCACTTAAGGTGGT **[-3]**
CG10089-m5
CGCGAAGGCAACGTACTCATCCACTGCCTG---------------------------------GCCTATATCATGACGGCCACACACC
TGAACTGGAAGGAGGCACTTAAGGTGGT **[-33]**

CG10426-wt
GCTGAGCAAGCGACCTAGTAAGGTGGAGCC……GCGATGGAGGAGCCGACGTGGTGATGCGGCGCAAGTC

CG10426-m1
GCTGAGCAAGCGACCTA---AGGTGGAGCC……GCGATGGAGGAGCCGACGTGGTGATGCGGCGCAAGTC **[-
3]**
CG10426-m2
GCTGAGCAAGCGACCTA---AGGTGGAGCC……GCGATGGAGGAGCCGACGatggttttagagctagaaatagcaagttaaa
ataaggctagtccgttatcaacttgaaaaagtggcaccgagtcggtgctagtcggTGCGGCGCAAGTC **[-3,+80]**
CG10426-m3
GCTGAGCAAGCGACCT--AAGGTGGAGGCC……GCGATGGAGGAGCCGACGTGGT-----GGCGCAAGTC **[-7]**
CG10426-m4
GCTGAGCAAGCGACCTAG-----TGGAGCC……GCGATGGAGGAGCCGACGTG-------GCGCAAGTC **[-13]**

CG10592-wt
ACTTCGAGGCCCTCGACGAGGAGCTGGACACACGATTCTGGCACGATAAGGCCCAATCGATTCTGGCCGAT
AAGCTGGCCGGCCATAAAAAGCTCAACGAGAATCGCGCCAAGAACGTGA
CG10592-m1
ACTTCGAGGCCCTCGACGAG--------------------GCACGATAAGGCCCAATCGATTCTGGCCGATAAGCTGGCCG
GCCATAAAAAGCTCAACGAGAATCGCGCCAAGAACGTGA **[-20]**
CG10592-m2
ACTTCGAGGCCCTCGACGAGGAGCTGGACA-----------CACGATAAGGCCCAATCGATTCTGGCCGATAAGCT
GGCCGGCCATAAAAAGCTCAACGAGAATCGCGCCAAGAACGTGA **[-11]**
CG10592-m3
ACTTCGAGGCCCTCGACGAGGAGCTGG---------------ACGATAAGGCCCAATCGATTCTGGCCGATAAGCTGG
CCGGCCATAAAAAGCTCAACGAGAATCGCGCCAAGAACGTGA **[-15]**
CG10592-m4
ACTTCGAGGCCCTCGACGAGGAGCTGGACACACGA---C---AC---
---AAGAACGTGA **[-72]**

CG11425-wt
TCTGCGGCCCCCCATCCGCCTCCTGGTGGACCTGGTCCTGCTGGGCCTACTTATTGTCCT
CG11425-m1
TCTGCGGCCCCCCATCCG---CCTGGTGGACCTGGTCCTGCTGGGCCTACTTATTGTCCT **[-3]**

CG11426-wt
CGGCTCCACGGGACCGTCATCGGATCGCCGGATGACGCAGCGCCTGCTCGTGGAGCTCCT……GCCGGTA
ATGCTCGGCCTGATCGTGGGCCTGCTTCCGGCCCTGGTCATGGTGGTGGTGGA
CG11426-m1
CGGCTCCACGGGACCGTCATCGGATCGCCGGATGACGCAGCGCCTGCTCGTGGAGCTCCT……GCCGGTA
ATGCTCG------------GCCTGCTTCCGGCCCTGGTCATGGTGGTGGTGGA **[-12]**
CG11426-m2
CGGCTCCACGGGACCGTCATCGGATCGCCGGATGACGCAGCGCCTGCTCGTGGAGCTCCT……GCCGGTA
ATGCTCG---------TGGGCCTGCTTCCGGCCCTGGTCATGGTGGTGGTGGA **[-9]**
CG11426-m3
CGGCTCCACGGGACCGTCATCG--------GATGACGCAGCGCCTGCTCGTGGAGCTCCT……GCCGGTAATGC
TCGGCCTGATCGTGGGCCTGCTTCCGGCCCTGGTCATGGTGGTGGTGGA **[-8]**

CG11437-wt
TCTTCGACCCGCTCAATCTTCAGTGTGAAAATGTGCGGCAATCCGAACACGCGTCTCCTC……TATCCCTAT
CGCCAGCCCTGGCTGACCAAGGTCCACCTTACGATCGCAGTGGTTGCTCTG
CG11437-m1
TCTTCGACCCGCTCAATCTTCAGTGTGAAAATGTGCGGCAATCCG------CGTCTCCTC……TATCCCTATCGC
CAGCCCTGGCTGACCAAGGTCCACCTTACGATCGCAGTGGTTGCTCTG **[-6]**
CG11437-m2
TCTTCGACCCGCTCAATCTTCAGTGTGAAAATGTGCGGCAATCCGAt---GCGTCTCCTC……TATCCCTATCG
CCAGCCCTGGCTGACCAAGGTCCACCTTACGATCGCAGTGGTTGCTCTG **[-4,+1]**
CG11437-m3
TCTTCGACCCGCTCAATCTTCAGTGTGAAAATGTGCGGCAATCCGAACACGCGTCTCCTC……TATCCCTAT
CGCCAGCC---GCTGACCAAGGTCCACCTTACGATCGCAGTGGTTGCTCTG **[-3]**
CG11437-m4
TCTTCGACCCGCTCAATCTTCAGTGTGAAAATGTGCGGCAATCCGAACACGCGTCTCCTC……TATCCCTAT
CGCCAGCCa--GCTGACCAAGGTCCACCTTACGATCGCAGTGGTTGCTCTG **[-3,+!]**

CG11438-wt
GTCCAAGTATTCGAAGCTGGCCCGCGGGTTCTGCGACCTGCTGATCTGGGTGGCCCTCAGCGTGGCCAGT
GTGCTGCTCCACAAGATGGGGCGTCCCTTCCGGCGCGGCTTCTTCTGTGG
CG11438-m1
GTCCAAGTATTCGAAGCTGGCCCGCGGGTTCTGCGACCTGCTGATCTGGGTGGCCCTCAGCGTGGCCAGT
G------------------GGCGTCCCTTCCGGCGCGGCTTCTTCTGTGG **[-18]**
CG11438-m2
GTCCAAGTATTCGAAGCTGGCCCGCGGGTTCTGCGACCTGCTGATCTGGGTGGCCCTCAGCGTGGCCAGT
GTGCTGCTCCACAcAcTGGaGCGTgaag-CCGGCGCGGCTTCTTCTGTGG **[-8,+7]**
CG11438-m3
GTCCAAGTATTCGAAGCTGGCCCGCGGGTTCTGCGACCTGCT-------------------GTGGCCAGTGTGCTGCTCCA
CAAGATGGGGCGTCCCTTCCGGCGCGGCTTCTTCTGTGG **[-19]**

CG11597-wt
GGTCAGTTCGAGGATCTGCTCCACCTGCTGGAATTGGGCGGTTCGGTGCAGGAGCATCGC
CG11597-m1
GGTCAGTTCGAGGATCTGCTCCACCTGCTGGAATTGGGCGGTTCG-TGCAGGAGCATCGC **[-1]**

CG17027-wt
ACTTTCTCATGGAGAAAATATTGGCCAGATACCCGGATCACAAGTTCATCGGCGAGGAGG
CG17027-m1
ACTTTCTCATGGAGAAAATATTGGCCAG--------ATCACAAGTTCATCGGCGAGGAGG **[-8]**
CG17027-m2
ACTTTCTCATGGAGAAAATATTGG-------CCCGGATCACAAGTTCATCGGCGAGGAGG **[-7]**

CG17028-wt
TGCGGTCCGCTC…………………………………………………………………………………ATCATAGATCCCA
CG17028-m1
TGCGGTCCGCTC---……ATGGGACCGGTCCGGTCATC……---ATCATAGATCCCA **[-181]**

CG17029-wt
TTCTACGACCTGGTGACCGTGTACGATAAGCAAATCGAGGATATTTTGACCGAGGGATTGGTGGCCGCCTTT
CCCGAATCCCTGATCATTGGCGAGGAGGAGTCGGCAGTTTCAC
CG17029-m1
TTCTACGACCTGGTGACCGTGTACGATAAGCAAATCGAGGATATTTTGACCGAGG------TGGCCGCCTTTCCC
GAATCCCTGATCATTGGCGAGGAGGAGTCGGCAGTTTCAC **[-6]**
CG17029-m2

TTCTACGACCTGGTGACCGTGTACGATAAGCAAATCGAGGATATTTTGACCGAGGtggccGATTGGTGGCCGC
CTTTCCCGAATCCCTGATCATTGGCGAGGAGGAGTCGGCAGTTTCAC **[+5]**
CG17029-m3
TTCTACGACCTGGTGACCGTGTACGATAAGCAAATCGAGGATATTTTGACCGAGGcA-------CCGCCTTTCCCG
AATCCCTGATCATTGGCGAGGAGGAGTCGGCAGTTTCAC **[-8,+1]**
CG17029-m4
TTCTACGACCTGGTGACCGTGTACGATAAGCAAATCGAGGATATTTTGACCGAGGtttgactGATTGGTGGCCG
CCTTTCCCGAATCCCTGATCATTGGCGAGGAGGAGTCGGCAGTTTC **[+7]**
CG17029-m5
TTCTACGACCTGGTGACCGTGTACGATAAGCAAATCGAGGATATTTTGACCGAG--ATTGGTGGCCGCCTTTC
CCGAATCCCTGATCATTGGCGAGGAGGAGTCGGCAGTTTCAC **[-2]**

CG17746-wt
CAACACACTCCACACCGCACACCATACAAGCCGCAATGGGTCAGACTCTATCGGAACCGGTGA……TGCAG
GGGTGGCGCATCAACATGGAGGACTCGCACACCCACATCCTCTCGCTGCCGGACG
CG17746-m1
CAACACACTCCACACCACACACCATACAAGCCGCAATGGGTCAGACTCTATCG------GTGA……TGCAGGGG
TGGCGCATCAA---------CTCGCACACCCACATCCTCTCGCTGCCGGACG **[-15]**
CG17746-m2
CAACACACTCCACACCGCACACCATACAAGCCGCAATGGGTCAGACTCTATCGGAACCGGTGA……TGC-----
TGG----------------ACTCGCACACCCACATCCTCTCGCTGCCGGACG **[-21]**
CG17746-m3
CAACACACTCCACACCGCACACCATACAAGCCGCAATGGGTCAGACTCTATCGGAACCGGTGA……TGCAG
GGGTGGCG------CATGGAGGACTCGCACACCCACATCCTCTCGCTGCCGGACG **[-6]**
CG17746-m4
CAACACACTCCACACCGCACACCATACAAGCCGCAATGGGTCAGACTCTATCGGAACCGGTGA……TGCAG
GGGTGGCGCATCA---------ACTCGCACACCCACATCCTCTCGCTGCCGGACG **[-9]**

CG5150-wt
GAGTCTTAAGTTCTGGATTCGCCTCGGTCTGCTGACCGTCGTTTGGGGTG……GAGGCGAGGAGTTGAAGC
TCTCCTTCGAAGAATTCCCCTTTACCGGACTATCGAAAACCT
CG5150-m1
GAGTCTTAAGTTCTGGATTCGCCTCGGTCTGCTGACCGTCGTTTGGGGTG……GAGGCGAGGAGTTGAAGC
TCTCCTTCGAAGAATTCCCC------CCGGACTATCGAAAACCT **[-4]**
CG5150-m2
GAGTCTTAAGTTCTGGATTCGCCTCGGTCTGCTGACCGTCGTCG------……---A
CCGGACTATCGAAAACCT **[-287]**
CG5150-m3
GAGTCTTAAGTTCTGGATTCGCCTCGGTCTGCTGACCGTCGTTTGGGGTG……GAGGCGAGGAGTTGAAGC
TCTCCTTCGAAGAATTCCCCTTTtccCCGGACTATCGAAAACCT **[+3,-1]**
CG5150-m4
GAGTCTTAAGTTCTGGATTCGCCTCGGTCTGCTGACCGTCGTTTGGGGTG……GAGGCGAGGAGTTGAAGC
TCTCCTTCGAA----------------------------AACCT **[-26]**

CG9238-wt
AAGCCATATATAAAATGATTTCGCACAGTCCGCCCATATTCAGTCACAGTCCACCCGTTA
CG9238-m1
AAGCCATATATAAAATGATTTCGCACAG---------------------TCCACCCGTTA **[-21]**

CG9389-wt
GGAAACGCGGCTACCCGATTCGGCTTGGAGGATCACGAGGCCACTCAGCAGGATGCCCGGGTGAACAC
A……GTCATCGGAGGAATCAGAGAAACGCGTCCCACCTGCTGCTGCATCGGCGCCTCCTCCAGC
CG9389-m1
GGAAACGCGGCTACCCGATTCG-------------------------GCAGGATGCCCGGGTGAACACA……GTCATCGGAGG
AATCAGAGAAACGCGTCCCACCTGCTGCTGCATCGGCGCCTCCTCCAGC **[-25]**
CG9389-m2
GGAAACGCGGCTACCCGATTCGGCTTGGAGGATCACGAGGCCACTCAGCAGGATGCCCGGGTGAACAC
A……GTCATCGGAGGAATCAGAGAAACGCGTCCCACCT------GCATCGGCGCCTCCTCCAGC **[-6]**

CG9391-wt
ACAACGTGGACGTGGAAAAGTGCTTAGAGGTGGCCAGCAACCTGGTTTCCGAAGCCGGAA
CG9391-m1
ACAACGTGGACGTGGAAAAG-------AGGTGGCCAGCAACCTGGTTTCCGAAGCCGGAA **[-7]**
CG9391-m2
ACAACGTGGACGTGGAAAAG---------GTGGCCAGCAACCTGGTTTCCGAAGCCGGAA **[-9]**

CG9449-wt
CACCCGACGGCATTGTCTCGCGATGACGGGCGGACTAATTGCATCGGCGGTCATAATCTG
CG9449-m1
CACCCGACGGCATTGTCTCGCGATGACGGGCGGACTAATT------GCGGTCATAATCTG **[-6]**
CG9449-m2
CACCCGACGGCATTGTCTCGCGATGACGGGCGGACTAATT----CGGCGGTCATAATCTG **[-4]**
CG9449-m3
CACCCGACGGCATTGTCTCGCATTGTCGG------------------CGGTCATAATCTG **[-18]**

CG9451-wt
GAAGTGCCGCAGG AAGTCACGGAAGGTCCTGGGGATGTCTCTCAAAGCTCAGC……ACCTACGAGCCCTT
CGGTTGGGGAGCACTCACCAATGTTAGTACTTATCAAACATAAACAAGACTATTATCGAAGCTA
CG9451-m1
GAAGTGCCGCAGGAAG------------------------------------……---------------------------TACATCACTGTTAGTACTTATC
AAACATAAACAAGACTATTATCGAAGCTA **[-1001]**
CG9451-m2
GAAGTGCCGCAGGAAG------------------------------------……---------------------------TACGTCACTGTTAGTACTTATC
AAACATAAACAAGACTATTATCGAAGCTA **[-1001]**
CG9451-m3
GAAGTGCCGCAGGcAAGTCACtcAAaGcTgatGtGATGTCTCTCAAAGCTCAGC……ACCTACGAG----------------C
ACTCACCAATGTTAGTACTTATCAA-CAT--AACAGACTATTATCGAAGCTA **[-28,+9]**

CG34140-wt
TCTATACAGTCATATAGTAACCGTAACTTTCAGAACAGACGCCGGTGTACACGCCCTGCA
CG34140-m1
TCTATACAGTCATATAGTAACCGTAACTTTCAGAACA--------------CGCCCTGCA **[-14]**

CG5946-wt
GTGCCACTCGCCGTGGGTGTGGTAGCCGTACTCGCCGGCGCCCTGATCGTCCACTATCTGCTGAATAAGAA
GTCCACGAAACCACGCCGGGAACCCAATCGCACCGCTCGGCTGCGCACACTTGTGGATCCCAATGA
CG5946-m1
GTGCCACTCGCCGTGGGTGTGGTAGCCG---------GCGCCCTGATCGTCCACTATCTGCTGAATAAGAAGTCC
ACGAAACCACGCCGGGAACCCAATCG-----CTCGGCTGCGCACACTTGTGGATCCCAATGA **[-14]**

CG10177-wt
TGCGGGTGTGCTTCCTGCGGAACGGAGATAGACACTTCAAGGGCGTTAATTTGGTCATCT
CG10177-m1
TGCGGGTGTGCTTCCT--------GAGATAGACACTTCCAGGGCGTTAATTTGGTCATCT **[-8]**
CG10177-m2
TGCGGGTGTGCTTCCTG--GAACGGAGATAGACACTTCAAGGGCGTTAATTTGGTCATCT **[-2]**
CG10177-m3
TGCGGGTGTG------------CGGAGATAGACACTTCCAGGGCGTTAATTTGGTCATCT **[-12]**

CG12069-wt
AGCAAGCGCAGATGCACTTCAGTCCCAAGGTGGACTACATTCTCATCCTGGACAAGTTGC
CG12069-m1
AGCAAGCGCAGATGCACTTCAGTCCCAAGGTGGACTACATTCTC---CTGGACAAGTTGC **[-3]**
CG12069-m2
AGCAAGCGCAGATGCACTTCAGTCCCAAGGTGGACTACATTCTCA---------AGTTGC **[-9]**

CG14305-wt
GAAGTTCGGATGTGGATGCACTGGCACAGCGGGGCTACAATGTGGGTCACAAGATCGGCGAGGGGTCTTA
TGCCACTGTTATAACCGCCGGTTATGCCGATGATCATGGACATGGAGTAC
CG14305-m1
GAAGTTCGGATGTGGATGCACTGGCACAGCGGGGCTACAATGTGGGTCACAAGATC--CGAGGGGTCTTATG
CCACTGTTATAACCGCCGGTTATGCCGATGATCATGGACATGGAGTAC **[-2]**
CG14305-m2
GAAGTTCGGATGTGGATGCACTGGCACAGCGGGGCTACAATGTGGGTCACAAGATCG-----GGGTCTTATGC
CACTGTTATAACCGCCGGTTATGCCGATGATCATGGACATGGAGTAC **[-5]**

CG1951-wt
TGACCGGGAGACCATGCTGGAAACACTGCGTCGCGGGGTTCAACAGCTAACAAAAATTCG
CG1951-m1
TGACCGGGAGACC----TGGAAACACTGCGTCGCGGGGTTCAACAGCTAACAAAAATTCG **[-4]**
CG1951-m2
TGACCGGGAGACC---CTGGAAACACTGCGTCGCGGGGTTCAACAGCTAACAAAAATTCG **[-3]**
CG1951-m3

TGACCGGGAGAC-----TGGAAACACTGCGTCGCGGGGTTCAACAGCTAACAAAAATTCG **[-5]**

CG2246-wt
ACTGGTGACCTTCTTCTGCTCACTAATTATATGTCTTTTACCCCCAAAAGGGATGCCAACAATAATATCATGG
AGCTTCTGATTATGGCCTACGCCTGCAAGACTTCCTCGGCTCGTTCG
CG2246-m1
ACTGGTGACCTCCTTCTGCTCATTAATTATATCTCTTTTACCCCCAAAGa-GATGCCAACAATAATATCATGGA
GCTTCTGATTATGGCCTACGCCTGCAAGACTTCCTCGGCTCGTTCG **[-2,+1]**
CG2246-m2
ACTGGTGACCTCCTTCTGCTCATTAATTATATCTCTTTTACCCCCAAAGa-GATGCCAACAATA---TCATGGAG
CTTCTGATTATGGCCTACGCCTGCAAGACTTCCTCGGCTCGTTCG **[-5,+1]**

CG31431-wt
TGTGCGGCTTTCAGCAGTCCCGCTGTTTTTTTGTTGACTATACAGGTGAGTTTAGTTCTTA
CG31431-m1
TGTcCctGGCTTTCAGCTGTCCCACTGTTTTTTTGTTGACTATACAGGTGAGTTTAGTTCTTA **[+3,-1]**

CG3809-wt
CTACCAGAGGGAATACTTATGGGCTTTGGCAACCCCCTGCTGGACATCACCTGCACCGTTGAGGATAATGT
GATCCTGGA
CG3809-m1
CTACCAGAGGGAATACTTATGGGCTTTGGCAACCCCCTGCTGGACATCACCTGCAC--TTGAGGATAATGTGA
TCCTGGA **[-2]**

CG10999-wt
CGCACGCAACAGGAGCAGGAGGTGCGTAGGCGGGAGCTGATGAGCACGAAGTCAAGTGCC
CG10999-m1
CGCACGCAACAGGAGCAGGAGGTGCG------GGAGCTGATGAGCACGAAGTCAAGTGCC **[-6]**
CG10999-m2
CGCACG--------------------------GGAGCTGATGAGCACGAAGTCAAGTGCC **[-26]**

CG12538-wt
GGTGGCTCTGGCGTGCTATTTGAATTGAACAAGTGTCAGCGTCGAGGGGAAACAGCGTCGAAGCTGACGCT
CCATGGTGCTCCAATAAAACTACCAAAGCCCCCTCCCGCCG……GAAACACAAATACTAGACGGC
CG12538-m1
GGTG----------------------------GTGTCGGTG---------------------------------------TCCAATAAAACTACCAAAGCCCCCTC
CCGCCG……GAAACACAAATACTAGtgtagctcttttggccgcgaC **[-67,+16]**
CG12538-m2
GGTGGCTCTGGCGTGCTATTTGAATTGAACAAGTGTCAGCGTCGAGGGGAAACAGCGTCGAAGCTGACGCT
CCATGG-----------------------CCCCTCCCGCCG……GAAACACAAATACTAGtgtagctcttttggccgcgaC **[-23,+16]**
CG12538-m3
GGTGGCTCTGGCGT--GCTCCAATAAAACTACCAAAGCCCCCT
CCCGCCG……GAAACACAAATACTAGtgtagctcttttggccgcgaC **[-64,+16]**
CG12538-m4
GGTGGCTCTGGCGTGCTATTTGAATTGAACAAGTGTCAGCGTCGAGGGGAAACA------------------------------------
--------GCCCCCTCCCGCCG……GAAACACAAATACTAGtgtagctcttttggccgcgaC **[-44,+16]**

CG12746-wt
CGGGGAGCTGCTCTTCTGGGTGATAGTGGCTCCCTTTCTGGTCACTATCGCCTTCTACTG
CG12746-m1
CGGGGAGCTGCTCTTCTGGGTGA--------------------ACTATCGCCTTCTACTG **[-20]**

CG14297-wt
CTTTGCCATTTTGCATGAAAAACTTACGTCATTTCGAAGGCAACTCGTGCAGCTCTCCGATGGCCGAGGTGA
TTATGCAGAATCTGATGGTCAAGACGAGTCTCTACTGGGAGGTGGATAGTGCCGGTCTGAGGACATGGAAC
ACT
CG14297-m1
CTTTG--AT---GCAaGAAAAACTTccATCATTTCGAAGGCAACTCGTGCAGCTCTCCGATGGCCGAGGTGATTAT
GCAGAAT--GATGGTCAAGACGAGTCTCTACTGGGAGGTGGATAGTGCCGGTCTCAGGACATGGAACtggacA
CT **[-10,+8]**
CG14297-m2
CTTTG--AT---GgAaGAAAAACTTccATCATTTCGAAGGCAACTCGTGCAGCTCTCCGATGGCCGAGGTGATTAT
GCAGAATCTGATGGTCAAGACGAGTCTCTACTGGGAGGTGGATAGTGCCGGTCTCAGGACATGGAACtggac
ACT **[-9,+9]**

CG2104-wt

GGCGTACTTTACACTGATCACGTACTTGAACGATGTGAGCGCCGAGATACAGGGAATCCG
CG2104-m1
GGCGTACTTTACACTGATCACGTACTTGAACGATGTGAGCGC--------AGGGAATCCG **[-8]**

CG31469-wt
CACATGCCGGTCACCAATGGCCGAGGCCATACTGAAGCATTTGGTAGTGAAACGGAATCTGCAGGACTGGT
ATGTGGACAGTGCTGGCCTCAGGAGTTGGAACGTTGGCCTGGAGCCCC
CG31469-m1
CACATGCCGGTCACCAATGGCCGAGGCCATACTGAAGCATTTGGTAGTGAAACGGAATCcTGCAGGACTGG
TATGTGGACAGTGCTGGCCTCAGGAGTTGGAACGTTGGCCTGGAGCCCC **[+1]**

CG5361-wt GCAATGGTAACCACACAGCAGACCAAGGAGATTTGCGAAATTATTCGGCTGAAATCAAC
CCAGGTGTTCC……AGGTGGCGACCTCCCAGAAAATCT……AATCAGCAGGATTGCGGAAGGTTATTTAATG
CAACGCAAGGCCCGATAAGCA
CG5361-m1
GCAATGG--……-----CGACCTCCCAGAAAATCT……AATCAGCA
GGATTGCGGAAGGTTATTTAATGCAACGCAAGGCCCGATAAGCA **[-189]**
CG5361-m2
GCAATGGTAACCACACAGCAG--……--GTGGCGACCTCCCAGAAAATC
T……AATCAGCAGGATTGCGGAAGGTTATTTAATGCAACGCAAGGCCCGATAAGCA **[-171]**
CG5361-m3
GCAATGGTAACCACACAGCAGAGAAGTGTGGTTAAC-----------------------------------……------------------------……----
--CTTCCTTGCGGAAGGTTATTTAATGCAACGCAAGGCCCGATAAGCA **[-468]**

CG6036-wt
AGTCATCCATGAGTGAGTGGGATTTCTGTGTG……TCATGGGAATGGGCTGCGATACTGCGTAAGTTCTATG
CAAGGTTGGCGATTGGAAATGGAGGATAGCCACTCGGCTGCTTGCCGGCTGAAGGATCCCTTCGCAACGT
CG6036-m1
AGTCATCCATGAGTGAGTGGGgagtggggATTTCTGTGTG……TCATGGGAATGGGCTGCGATACTGCGTAAGT
TCTATGCAAGGTTGGC-AccTT-------------CCACTCGGCTGCTTGCCGGCTGAAGGATCCCTTCGCAACGT **[+10, -16]**

CG13369-wt
TAACAGACTACCCACAAGAGACTAGTGCAATTGCAGCAATGGCCCAAACGGAAGTGCTGGTGTTCGGCTCG
GCCATCATTGACTTTATAAGGTAGGTGGGCAGAGAAGGCTCGATCCCAA
CG13369-m1
TAACAGACTACCCACAAGAGACTAGTGCAATTGCAGCAATGGCCCAAACGGAAGTGCTGGTGTT--GCTCGG
CCATCATTGACTTTATAAGGTAGGTGGGCAGAGAAGGCTCGATCCCAA **[-2]**
CG13369-m2
TAACAGACTACCCACAAGAGACTAGTGCAATTGCAGCAATGGCCCAAACGGAAGTGCT-----------CGGCCATC
ATTGACTTTATAAGGTAGGTGGGCAGAGAAGGCTCGATCCCAA **[-11]**
CG13369-m3
TAACAGACTACCCACAAGAGACTAGTGCAATTGCAGCAATGGCCCAAACGGAAGTGCTGGTGT---GCTCGGC
CATCATTGACTTTATAAGGTAGGTGGGCAGAGAAGGCTCGATCCCAA **[-3]**

CG2577-wt
TCCACAGTGGGGAGCGGGTGGCCATCAAGGTGGAAAGCAGCAAGGTGCGCCATCCGCAGCTCAACTA……
AGGCCCGCCCACGGTTTGCCAAGGATTAGGTACTTCCACAAGGAGGAGCACTACCAGGCGATGGTTATGGA
TCTGC
CG2577-m1
TCCACAGTGGGGAGCGGGTGG-------GG--GA--G-----------------------------------……---
------------CGATGGTTATGGATCTGC **[-124]**

CG32649-wt
GGAATCGGCCATCTCTGAGCCTGCACAAGTCTCCTCAATTCGTAAATCCACACC
CG32649-m1
GGAATCGGCCATCTCTGAGCCTGCACAAGTCTCCTCAAgTcctccTCGTAAATCCACACC **[+6]**

CG32687-wt
AGGAGAAGATCAGCCATGGAGGTGTACACGTCGGACAGTTCGGACACGGATTCGCGGGAGCAGAAGACAC
TGGACTTTGGCCGTATGAGCCTGGACCTGGTCACGCTGGAGGATCACCTGGCCTCGCCGCAGAAGGCTCT
CG32687-m1
AGGAGAAGATCAGCCATGGAGGTGTACACG---CTGGA
GGATCACCTGGCCTCGCCGCAGAAGGCTCT **[-75]**
CG32687-m2

AGGAGAAGATCAGCCATGGA--CACGCTGGtGGAT
CACCTGGCCTCGCCGCAGAAGGCTCT **[-82,+1]**
CG32687-m3
AGGAGAAGATCAGCCATGGAGGTGTACACG---CCTGGACCTGGT
CACGCTGGAGGATCACCTGGCCTCGCCGCAGAAGGCTCT **[-59]**
CG32687-m4
AGGAGAAGATCAGCCATGGAGGTGTACACGTCGGACtgtAcacGTTCGGACACGGATTCGCGGGAGCAGAAG
ACACTGGACTTTGGCCGTAT-AGCCTGGACCTGGTCACGCTGGAGGATCACCTGGCCTCGCCGCAGAAGGC
TCT **[+6,-1]**

CG4041-wt
AATTCCACACGTGCCAAGATTACAGATTCATCTACGGATAACACTCACTAATTCCACGACTGCC……GAGTAT
CTGGGACTCTCACTGGAGGACTACGCCATGCGTCATCCACCGCTGGCCATTGC
CG4041-m1
AATTCCACACGTGCCAAGATCACAGATTCATCTACGGATAACACTCACTAATTCCA-G--TGCC……GAGTATC
TGGGACTagTCACTGGAGGACTACGCCATGCGTCATCCACCGCTGGCCATTGC **[-4,+2]**
CG4041-m2
AATTCCACACGTGCCAAGATCACAGATTCATCTACGGATAACACTCACTAATTCCA-G--TGCC……GAGTATC
TGGGA---TCACTGGAGGACTACGCCATGCGTCATCCACCGCTGGCCATTGC **[-6]**

CG8173-wt
ATCAACGTGCCGCCATCGCCGATGATGAAGACCCTGGGCCACGGCACTGGGATCCGGGTCTACCGCTTGG
ACCGCTCGCCTCGTCTCGGCGAAATCCGCTCGCCCTGGGCCGTCAAG
CG8173-m1
ATCAACGTGCCGC--TCGCCTCGTCTCGGCGAAATCCGCTCGC
CCTGGGCCGTCAAG **[-62]**
CG8173-m2
ATCAACGTGCCGCCATCGCCGATGATGAAGACCCTGGGCCACGGCACTGGGA-------------------CCGCTCGCC
TCGTCTCGGCGAAATCCGCTCGCCCTGGGCCGTCAAG **[-19]**
CG8173-m3 ATCAACGTGCCGCCATCGCCGATGATGAAGACCCTGGGCCACGGCACTGGGATCCGGG
TCTACCGCTTGGACCGCTCGCCTCGTCTCGGCGAAATCCGCTCGCCCTGGGCCGTCgcctcgtctcggcgaaatccg
ctcgccctggAAG **[+31]**
CG8173-m4
ATCAACGTGCCGCCATCGCCGATGATGAAGACCCTGGGCCACGGCACTGGGATCCGGGTCTAC---TTGGAC
CGCTCGCCTCGTCTCGGCGAAATCCGCTCGCCCTGGGCCGTCAAG **[-3]**

CG8565-wt
GCTCTCCGATGATGGAATTGCCGGCAGAAAGCCGGCCAAAATTAATAAGTATAGCGAACTTAGC
CG8565-m1
GCTCTCCG--------------------------GCCAAAATTAATAAGTATAGCGAACTTAGC **[-26]**
CG8565-m2
GCTCggaGATGAT----TcGCCG--Attc-GCCGGCCAAAATTAATAAGTATAGCGAACTTAGC **[-14,+7]**
CG8565-m3
GCTCTCCGATGA------------------GCCGGCCAAAATTAATAAGTATAGCGAACTTAGC **[-18]**

CG15743-wt
TCCATCAGGAGGAGCGGCCAGCGATCTACGGCATGCTGCGCAGTGAGAATCCCAGCCGCG
CG15743-m1
TCCATCAGGAGGAGCGGCCAGCGA-CTACGGCATGCTGCGCAGTGAGAATCCCAGCCGCG **[-1]**

CG1637-wt
ATCTGTCCTTTGGAGGTGAGTGCGATCTTCTTTACTTTTCGCATTATGTAAATTGCTCAC
CG1637-m1
ATCTG--------AGGTGAGTGCGATCTTCTTTACTTTTCGCATTATGTAAATTGCTCAC **[-8]**
CG1637-m2
ATCT-----TTGGAGGTGAGTGCGATCTTCTTTACTTTTCGCATTATGTAAATTGCTCAC **[-5]**

CG17598-wt
GTGCGGCCCATCATCGTGCCGCGGGACATCAACCTACTGCCCTGGGGCACCGGATACGCC
CG17598-m1
GTGCGGCCCATCATCGTGCCGCGGGACATCAACCTACTG-----GGGCACCGGATACGCC **[-5]**
CG17598-m2
GTGCGGCCCATCATCGTGCCGCGGGAC-------------CCTGGGGCACCGGATACGCC **[-13]**

CG32812-wt
TCGTCAGCTCAACCCCGTCCAACTGTGTGACCACCAGCAGGCCACAGGGCTGTCCGC
CG32812-m1

TCGTCAGCTCAACCgtGTtgaCCAACTGTGTGACCACCAGCAGGCCACAGGGCTGTCCGC **[-2,+5]**

CG32812-wt
CCTACGTTCCATTGGGATTAATCGAATCCGTACAGGTGCGAGATCTGTTCCAGCTGATTG
CG32812-m1
CCTACGTTC-AcTGGGATTAATCGAATCCGTACAGGTGCGAGATCTGTTCCAGCTGATTG **[-2,+1]**

CG42271-wt
TTGGTGTAAACTGCGTGGAAATTTACTGTTCTACCTCAAGGATAAGGACCCCAAGTCAGCGGTGGCCGGCC
TCCTGGTGCTGGAGAACTGTCGTGCGCGCATTCAGAATGAGGAGCGTGA
CG42271-m1
TTGGTGTAAACTGCGTGGAAATTTACTGTTCTACCTCAAGGATAAGGACCCC----CAGCGGTGGCCGGCCTC
CTGGTGCTGGAGAACTGTCGTGCGCGCATTCAGAATGAGGAGCGTGA **[-4]**
CG42271-m2
TTGGTGTAAACTGCGTGGAAATTTACTGTTCTACCTCAAGGATAAGGACCC----------------GGCCTCCTGGTGC
TGGAGAACTGTCGTGCGCGCATTCAGAATGAGGAGCGTGA **[-16]**
CG42271-m3
TTGGTGTAAACTGCGTGGAAATTTACTGTTCTACCTCAAGGAT---T
CAGAATGAGGAGCGTGA **[-59]**

CG7378-wt
AACAGACCACAGGCCGCCAGCTGCAGCGAGTCCTGCACTACTCGATGGCTCCTAGCCG……TTGATGGGCA
TCACCCACGTTCTGAACGCGGCCGAGGGATGTCGGTATGGTCAGGTGGAC
CG7378-m1
AACAGACCACAGGCCGCCAGCTGCAGCGAGTCCTGCACTACTCGATGGCTCCTAGCCG……TTGATGGGCA
TCACCCACGTTCTGAACGCGGCCGAGGGATGTCGGTATGGT-----GGAC **[-5]**
CG7378-m2
AACAGACCtccCAGGCCGCCAGCTGCAGCGAGTCCTGCACTACTCGATGGCTCCTAGCCG……TTGATGGGC
ATCACCCACGTTCTGAACGCGGCCGAGGGATGTCGGTATGG--------AC **[-9,+3]**

CG9784-wt
TCCGTGGACGCACAAGGCTAAGGAACTGCTACGCAACTACGACTATGTGGCCGTAAAGAC
CG9784-m1
TCCGTGGA------AG--TAAGGAACTGCTACGCAACTACGACTATGTGGCCGTAAAGAC **[-8]**

Supplementary Note 2. Detailed sequences for abnormal lethal events.

Note: wt, wild-type DNA/protein sequence; m#, mutation line# DNA/protein sequence. Deletions are shown as red dashes and insertions as highlighted in blue and lowercase letters. The change of DNA length (in nucleotides) caused by each mutation is indicated as purple (+, insertion; -, deletion). Note that some alterations have both insertions and deletions of nucleotides and in these cases the alterations are enumerated in the brackets. The DNA sequences are followed by the corresponding protein sequences. All DNA/protein sequences were obtained from the flybase website (flybase.org) and the alignments were performed by CLUSTALW.

CG12229-wt DNA sequence
ATG……GGACACCATGAACATTGCGATATCGGCGCATTCTGCAGAGAGGGAACTACGTCTGACAACTGGCT
TGGCTCTGGAGATTAACGGCGAGTGCTGTCGCGTTGGGAGACTGAGGAACAACTGCACC
CG12229-m1(viable) DNA sequence
ATG……GGACACCATGAACATTGCGATATCGGCGCATTCTGCAGAGAGGGAACTACGTCT----ACTGGCTTG
GCTCTGGAGATTAACGGCGAGTGCTGTCGCGTTGGGAGACTGAGGAACAACTGCACC **[-4]**
CG12229-m2(lethal) DNA sequence
ATG……GGACACCATGAACATTGCGATATCGGCGCATTCTGCAGAGAGGGAACTACGTCTGg--AACTGGCTT
GGCTCTGGAGATTAACGGCGAGTGCTGTCGCGTTGGGAGACTGAGGAACAACTGCAC **[-3,+1]**

CG12229-wt protein sequence
MPINQKEVTPQLDSQTGEDQGEEEEQPEDENRLEKFTVRLNGQHDLEQRLKDFKAACEAHEKHARRRKRKRYY
HLGLVAKLVTETTHDELRRMLENGTYTFHVDTVGNKPDELKAILDTMNIAISAHSAERELRLTTGLALEINGECCRV
G……
CG12229-m1(viable) protein sequence
MPINQKEVTPQLDSQTGEDQGEEEEQPEDENRLEKFTVRLNGQHDLEQRLKDFKAACEAHEKHARRRKRKRYY
HLGLVAKLVTETTHDELRRMLENGTYTFHVDTVGNKPDELKAILDTMNIAISAHSAERELRLLAWLWRLTASAVAL
GD*
CG12229-m2(lethal) protein sequence
MPINQKEVTPQLDSQTGEDQGEEEEQPEDENRLEKFTVRLNGQHDLEQRLKDFKAACEAHEKHARRRKRKRYY
HLGLVAKLVTETTHDELRRMLENGTYTFHVDTVGNKPDELKAILDTMNIAISAHSAERELRLELAWLWRLTASAVA
LGD*

CG17027-wt DNA sequence
AGTAAAAACGTCTCAATCAAAGGCGATTTCTACGACGTGGTCACCGACTACGACAACAAGATTGAGGACTTT
CTCATGGAGAAAATATTGGCCAGATACCCGGATCACAAGTTCATCGGCGAGGAGGAGACGGCTAAGAACAA
CAACGTGT
CG17027-m1(viable) DNA sequence
AGTAAAAACGTCTCAATCAAAGGCGATTTCTACGACGTGGTCACCGACTACGACAACAAGATTGAGGACTTT
CTCATGGAGAAAATATTGGCCAGAT--------CACAAGTTCATCGGCGAGGAGGAGACGGCTAAGAACAACAAC
GTGT **[-8]**
CG17027-m2(lethal) DNA sequence
AGTAAAAACGTCTCAATCAAAGGCGATTTCTACGACGTGGTCACCGACTACGACAACAAGATTGAGGACTTT
CTCATGGAGAAAATATTGGCCAGAT--------CACAAGTTCATCGGCGAGGAGGAGACGGCTAAGAACAACAAC
GTGT **[-8]**

CG17027-wt protein sequence
MAGVSQADIEELYNFIHPLAIKAGEILMEGYEMASKNVSIKGDFYDVVTDYDNKIEDFLMEKILARYPDHKFIGE
E……
CG17027-m1(viable) protein sequence
MAGVSQADIEELYNFIHPLAIKAGEILMEGYEMASKNVSIKGDFYDVVTDYDNKIEDFLMEKILARSQVHRRGGDG*
CG17027-m2(lethal) protein sequence
MAGVSQADIEELYNFIHPLAIKAGEILMEGYEMASKNVSIKGDFYDVVTDYDNKIEDFLMEKILARSQVHRRGGDG*

CG17028-wt DNA sequence
AGGAAGTGCGGTCCGCTC……ATCATAGATCCCATTGATGGCA
CG17028-m1(lethal) DNA sequence
AGGAAGTGCGGTCCGCTC---……ATGGGACCGGTCCGGTCATC……---ATCATAGATCCCATTGATGGCA **[-1,**
81]
CG17028-m2(lethal) DNA sequence
AGGAAGTGCGGTCCGCTC……ATCATAGATCCCATTGATGGCA **[0]**

CG17028-wt protein sequence
MSYRIGEEKLEVYYQVSLELVRKCGPLFLEGFQKPKTDYEVKSAFYDLVTVYDKQIEATLTDGLLKTFPESKIIGEE
AMANAKTPPELTDAPTWIIDPIDGTNNYVRKIPHCCISVGLAINKE……
CG17028-m1(lethal) protein sequence
MSYRIGEEKLEVYYQVSLELVRKCGPLMGPVRSSS*
CG17028-m2(lethal) protein sequence
MSYRIGEEKLEVYYQVSLELVRKCGPLFLEGFQKPKTDYEVKSAFYDLVTVYDKQIEATLTDGLLKTFPESKIIGEE
AMANAKTPPELTDAPTWIIDPIDGTNNYVRKIPHCCISVGLAINKE……
